# The impact of DRG reimbursement system on appropriate techniques of traditional Chinese medicine—evidence from pilot cities with traditional Chinese medicine hospitals in China

**DOI:** 10.3389/frhs.2025.1441482

**Published:** 2025-08-04

**Authors:** Jin Zhang, Yana Zhou, Dan Han, Chen Zhang, Yajuan Xiong

**Affiliations:** ^1^ Dong Fureng Institute of Economic and Social Development, Wuhan University, Wuhan, Hubei, China; ^2^ Medical Department of Hubei Hospital of Traditional Chinese Medicine, Wuhan, Hubei, China

**Keywords:** Diagnosis-Related Groups (DRG) reimbursement system, difference-in-difference method, appropriate techniques of traditional Chinese medicine (TCM), cost efficiency, policy impact

## Abstract

**Introduction:**

Given the ongoing development and significant medical role of Traditional Medicine Hospital (TCM) techniques in Chinese TCM hospitals, it is imperative to conduct an assessment on the influence of the gradual implementation of the DRG reimbursement system in W City's three public hospitals on the utilization of appropriate techniques of TCM.

**Methods:**

This study uses the Difference-in-Difference method to analyze the economic impact of the DRG reimbursement system. It compares cost changes between the TCM techniques group and other groups before and after DRG implementation to reveal the actual influence on cost. Visualization tools show cost variations from 2019 to 2022, bolstering research credibility. A T-test analyzes cost disparities, validating prediction model accuracy. Finally, a parallel trend test ensures reliability of the DID model's results.

**Results:**

(1)The total cost of appropriate techniques of TCM group decreased significantly (*p* < 0.001), with a 14.27% reduction in cost-effective TCM techniques (*p* < 0.001) but no significant changes in surgical cost.(2) Cost analysis of various TCM techniques showed significant decreases in total cost for external treatment and acupuncture, by 4.78% and 8.06% respectively (*p* < 0.001). cost for external treatment, orthopedic treatment, acupuncture, and special therapies also decreased by 13.67%, 14.27%, 16.8%, and 9.3% respectively (*p* < 0.001).(3)After analyzing 8 departments with high discharge rates, the total cost of TCM techniques in cardiology decreased by 18.86% (*p* < 0.001) under the DRG reimbursement system, while acupuncture cost increased by 11.85% (*p* < 0.001). In orthopedics, TCM techniques cost decreased by 30.3% (*p* < 0.001), but acupuncture and stomach/spleen departments saw significant increases of 18.88% and 46.66% respectively (*p* < 0.001).(4)Overall, there has been a significant reduction in the cost of TCM techniques following DRG payment reform. Notably, cost changes varied across departments, and acupuncture and moxibustion have experienced substantial cost fluctuations.

**Conclusions:**

The DRG payment reform has had a significant impact on appropriate techniques of TCM utilization and medical expense control, reflecting hospitals’ strategic adjustment in balancing service quality with the DRG policy. It is recommended that policymakers consider the compatibility of DRG with TCM methods to ensure fairness and efficiency.

## 1 Introduction

Faced with the persistent rise in global healthcare cost, governments worldwide are actively seeking effective measures to curb this trend and enhance the efficiency of healthcare services. Diagnosis-Related Groups (DRG), a proactive insurance payment model, have been widely adopted in numerous countries aiming to enhance transparency in healthcare services, improve efficiency, and rationally control expenditures (1–4). DRG classify diseases based on factors such as severity, treatment methods, and expected recovery time while setting fixed payment standards for each category. This system encourages healthcare institutions to optimize cost management and improve service quality by reducing unnecessary resource consumption (5, 6). Although DRG has promoted industry competition and medical innovation, its implementation also faces challenges such as the scientific validity of grouping, the need for stronger regulation, and policy adaptability (7–11).

In Chinese medical institutions, Western medicine and Traditional Chinese Medicine (TCM) complement each other, particularly in some TCM hospitals. For instance, appropriate techniques of TCM, grounded in TCM theories and traditional medical knowledge, are integrated with modern medical practices (12–15). These technologies are specifically designed for certain diseases or conditions. They employ treatment methods that are simple, effective, safe, and widely applicable. These include not only classical TCM treatments such as acupuncture, tuina (Chinese therapeutic massage), cupping, moxibustion, and Chinese herbal medicine, but also treatment plans that have been innovated and refined to suit specific populations or regions (16–18).

Appropriate techniques of TCM emphasize individualized treatment and offer several advantages, including notable clinical efficacy, operational simplicity, and low cost. These technologies are widely applied in the prevention and treatment of common and frequently occurring diseases, serving as an important complement to TCM within the modern healthcare system. For instance, in the field of orthopedics and traumatology, appropriate techniques of TCM have demonstrated irreplaceable value. Taking distal radius fractures—a common orthopedic condition—as an example, TCM employs unique manual reduction techniques combined with herbal therapies to achieve significant therapeutic outcomes (18–20). Compared with surgical interventions in Western medicine, appropriate techniques of TCM not only yield comparable efficacy but also provide distinct benefits in alleviating patient discomfort and reducing healthcare costs (19–21).

In the past decade, several experiments on DRG payment have been conducted on the mainland of China. However, the impact of DRG payment on healthcare has been mixed, with no systematic review of the relevant evidence (22–25).The literature generally indicates that DRG payment can improve healthcare efficiency by reducing hospitalization days (7, 25, 26). However, for hospitals specializing in Traditional Chinese Medicine(TCM), the implementation of the reimbursement system presents the following challenges (27–35).

Firstly, the design of the DRG system is primarily based on the treatment characteristics of Western medicine, which results in an incomplete reflection of the unique features and technical complexities associated with the treatments of TCM. Consequently, the appropriate techniques of TCM are disadvantaged in terms of cost accounting and payment standards, due to their exclusion from the surgical operation group within the DRG reimbursement system.

Secondly, the treatment protocols in TCM require a longer duration and personalized solutions, which do not align well with the DRG system's preference for short-term, standardized treatments. Consequently, this places Chinese TCM hospitals under dual pressures of cost and revenue.

Then, after the implementation of the DRG reimbursement system, there has been a reduction in the utilization of Chinese medicine, non-pharmacological therapies, and the treatment protocols in TCM.

Consequently, TCM hospitals encounter specific challenges in adapting to and implementing the DRG system, attributed to their unique diagnostic and therapeutic approaches, as well as distinct medical cultural characteristics. Particularly in employing the appropriate techniques of TCM, addressing the challenge of maintaining the unique characteristics of TCM while ensuring cost-effectiveness within the DRG system framework is a pressing issue.

In response to the challenges in the medical field, the Chinese government initiated a national pilot project for a DRG-based reimbursement system in 2017. The goal of this initiative is to develop a payment mechanism tailored to the unique conditions of domestic medical services. Following years of dedicated efforts, the National Medical Insurance Administration of China officially launched the “Three-Year Action Plan for DRG Payment Reform” in 2021. According to this plan, all eligible medical institutions, including TCM hospitals, are expected to fully implement the DRG payment method by the end of 2025 (27). The primary objective of this crucial reform measure for the medical insurance reimbursement system is to further facilitate the advancement of China's healthcare services towards superior quality, enhanced efficiency, and long-term sustainability (34, 35). However, a central challenge confronting this reform is adapting the reimbursement system to effectively incorporate Traditional Chinese Medicine (TCM) and its associated techniques. The current DRG payment model primarily draws on data derived from Western medicine treatment protocols, creating significant issues for accurately accounting for the unique characteristics and complexity of TCM therapies—such as acupuncture and tui-na (Chinese therapeutic massage).

Appropriate techniques of TCM,” as defined by the National Administration of Traditional Chinese Medicine, denote practices deeply rooted in TCM theory, rigorously validated through extensive clinical practice, and applicable to distinct medical conditions with proven safety, remarkable efficacy, and clear scalability. These essential techniques—including acupuncture, tui-na, cupping therapy, and moxibustion—are deeply grounded in traditional wisdom yet enhanced by contemporary medical and technological advances, effectively meeting the health needs of diverse populations. The effective implementation of these techniques within the DRG medical insurance reimbursement system is severely hampered by the absence of standardized operational definitions and reimbursement criteria. Prevailing inconsistencies in classifying and accounting for the costs of these TCM techniques under the DRG model have erected significant financial hurdles for both patients and TCM hospitals, undermining the equitable reimbursement of TCM treatments. It is therefore imperative to seamlessly incorporate these practices into the broader healthcare reimbursement framework by establishing clear, comprehensive guidelines and equitable payment structures that accurately reflect their unique characteristics, proven effectiveness, and substantial contribution to public health. This integration is crucial to ensure TCM maintains its indispensable role within China's robust healthcare system while actively promoting fairness, sustainability, and efficiency.

The objective of this study is to examine the economic impact of the DRG reimbursement system on the utilization of appropriate techniques of TCM, with a specific focus on cost containment and the optimization of treatment efficacy. While previous research has primarily concentrated on Western medical systems, it has often overlooked the unique treatment modalities and cost structures of Chinese medicine. Given the crucial role that TCM plays within China's healthcare system, understanding how DRG policies affect the operations of TCM hospitals and the provision of medical services is essential.

This study examines the impact of the DRG reimbursement system on the utilization of appropriate techniques of TCM, as well as their variations across different techniques. Adopting a statistical analysis methodology akin to that employed by Weiyan Jian in her analysis of inpatient expenses within DRG pilot cities in Beijing (7), this research conducts a comprehensive investigation of TCM treatment expense data from three public TCM hospitals in W City over the period from 2018 to 2022. The aim is to evaluate the influence of the DRG reimbursement system on the adoption of appropriate techniques of TCM.

The research findings indicate that, subsequent to the implementation of the DRG reimbursement system, there was a notable reduction of 4.68% in the total cost for groups using the appropriate techniques of TCM. Specifically, expenditures on TCM external treatments, orthopedic injuries, and acupuncture techniques decreased by 13.67%, 14.27%, and 16.80%, respectively. However, expenses related to surgical procedures did not exhibit significant changes. These outcomes suggest that TCM hospitals have strategically reduced their use of the appropriate techniques of TCM, aiming to balance the quality of medical services with adherence to DRG insurance payment policies. Consequently, it is imperative that policymakers consider the alignment of DRG insurance payment policies with the treatment of TCM modalities during their promotion in TCM hospitals. This alignment will ensure fairness and efficiency in medical insurance policies, while preserving the unique characteristics and benefits of the treatment of TCM.

The current literature has initiated an exploration into the economic impact of the DRG reimbursement system on the appropriate techniques of TCM (27–36). However, most studies have primarily focused on cost analysis and macro-level hospital operations, neglecting to fully address the specific impact on different appropriate techniques of TCM or departments in TCM hospitals. In comparison to existing research, this paper makes three significant contributions. Firstly, it provides empirical data on the specific application and cost analysis of appropriate techniques of TCM under the DRG reimbursement system, thereby addressing a gap in current research and enhancing understanding of how DRG influence the utilization of these techniques. Secondly, through a comprehensive analysis of DRG policy impacts, this study identifies varying effects across different departments and techniques, notably in treatments for bone injuries, external applications, and acupuncture within TCM. Furthermore, the findings reveal a decrease in the utilization of appropriate techniques of TCM among surgical patients, despite an increase in surgical cost, leading to an overall rise in total expenses. Lastly, these insights offer valuable implications for further comprehending and refining the implementation of the DRG reimbursement system in the realm of traditional Chinese medicine (41–44). This underscores the importance of balancing cost containment and quality of medical services while preserving the unique features and benefits of TCM.

The primary focus of this study centers on three public TCM hospitals in W City, China, utilizing data from the period 2018 to 2022. Consequently, the generalizability of the study findings might be limited by geographical and hospital selection biases. Furthermore, while the difference-in-differences method provides a robust approach for addressing potential confounders in time-series data, it is essential to recognize that no observational study can completely eliminate all sources of bias. Future research should aim to enhance the universality and robustness of these results by expanding the sample size and including a more diverse array of hospitals and regions.

## 2 Materials and methods

### 2.1 Data sources

W City, situated in the central region of China and serving as the provincial capital, not only serves as the core driver of economic development in the central region but also plays a pivotal role as a railway hub and logistics distribution center. Given its strategic significance and significant population mobility, ensuring the health and well-being of Chinese citizens through medical services provided by the city's healthcare institutions is of utmost importance. In January 2021, as a national reform pilot city, three public third-level A hospitals in W City fully implemented the CR-DRG medical insurance payment mechanism.

The present study collected comprehensive data on the utilization of the appropriate techniques of TCM from three public TCM hospitals between 2018 and 2022, encompassing a total of 32,154 cases. Following rigorous data screening procedures, a final sample size of 21,780 cases was determined. The dataset includes patients' basic information (such as age and gender), chronic disease status (including hypertension, diabetes, cerebral infarction, chronic obstructive pulmonary disease, and cancer), surgical operation details, itemized medical expenses, the appropriate techniques of TCM cost, as well as surgical expenditures. In defining the appropriate techniques of TCM for this study's purposes, we strictly adhered to the National Statistical System for Traditional Chinese Medicine Medical Services Management and the Guidelines for Filing Traditional Chinese Medicine Case Sheets. Consequently, six categories of TCM appropriate techniques were identified: TCM external treatment techniques; TCM orthopedic interventions; TCM acupuncture and moxibustion therapies; TCM massage techniques; TCM rectal and anal treatments; along with other related modalities within the realm of traditional Chinese medicine (36).

### 2.2 The characteristics of sample data

After a rigorous data selection process, we selected 21,780 patients as analysis subjects for this study. We examined patient and hospital characteristics during the stratification period of the DRG implementation from 2018 to 2022, as presented in Table 1. This table includes sociodemographic data such as hospitalization numbers, sex, and age distribution—where male patients increased from 45.6% to 58.7%, and the median age rose from 50 to 53 years—along with hospital characteristics across three tertiary care hospitals (A, H, and M) with consistent bed counts of 1,200, 1,500, and 1,350 respectively. Department classifications showed variable patient volumes, notably in acupuncture and moxibustion, gastroenterology, and proctology. Morbidity and surgical data indicated a slight rise in the Case Mix Index from 0.82 to 0.84, reflecting increased case complexity, while patient outcomes showed a decrease in in-hospital mortality from 0.1% to 0.07% and a reduction in average hospital stay from 10.49 to 9.4 days.

**Table 1:** Characteristics of hospitalized medical patients.

Variables	Before DRG implementation 2018–2020,NO.(%)	After DRG implementation 2021–2022,NO.(%)
Sociodemographic		
Hospitalization, No.	12428	9351
Male sex	5,676 (45.6)	3,862 (58.7)
Age, median (IQR)	50	53
0–18	470 (3.7)	274 (2.9)
19–45	3,737 (30.0)	3,322 (35.5)
46–60	3,517 (28.2)	2,529 (27.0)
>60	4,704 (37.8)	3,226 (34.4)
Hospital teaching level		
Tertiary care hospital(HospitalA/H/M)	12,428 (100)	9,351 (100)
Number of Bed		
Hospital A	1,200	1,200
Hospital H	1,500	1,500
Hospital M	1,350	1,350
Department classification(top %)		
Acupuncture and moxibustion	2,487 (20.0)	1,409 (15.0)
Gastroenterology	776 (6.2)	1,269 (13.5)
Proctology	1,322 (10.6)	1,008 (10.7)
Orthopedics	579 (4.6)	458 (4.8)
Cardiovascular internal medicine	627 (5.0)	552 (5.9)
Morbidity		
Chronic disease yes	4,103 (33.0)	2,793 (29.8)
Surgery yes	5,614 (45.1)	4,708 (50.3)
CMI	0.82	0.84
Patient outcomes		
In-hospital mortality	18 (0.1)	9 (0.07)
Length of hospital stay, mean (SD), d	10.49 (5.3)	9.4 (4.4)
Patient hospitalization cost		
Cost,mean (SD),yuan	13,777.2 (10,126.2)	14,017.6 (9,715.1)
Surgery_cost,mean (SD),yuan	2,448.9 (2,006.9)	2,512.4 (2,030.0)
TCM_tecnology_cost,mean (SD),yuan	3,032.2(3,782.7)	2,780.2(3,497.5）

Note: This table includes sociodemographic data such as hospitalization numbers, sex, and age distribution—where the percentage of male patients increased from 45.6% to 58.7%, and the median age rose from 50 to 53 years—along with hospital characteristics across three tertiary care hospitals (A, H, and M) with consistent bed counts of 1,200, 1,500, and 1,350 respectively.

Additionally, mean hospitalization cost saw a slight increase, particularly for surgery and appropriate techniques of TCM. Given the necessity of establishing pre- and post-policy implementation conditions for the experimental and control groups in the double difference method analysis, a meticulous selection and matching process was conducted, as detailed in Supplementary Figure S1. The experimental group, employing appropriate traditional Chinese medicine techniques, included 12,116 cases, and the control group comprised 9,663 cases (refer to Supplementary Table S2).

### 2.3 Study method

#### 2.3.1 The differences-in-differences (DID) method

In the field of health policy evaluation, the double difference method (DID) is widely recognized as a prominent research approach (27, 36–38). In this study, we employed a combination of the double difference model (DID) and high-dimensional fixed effect (HDFE) linear regression to conduct an in-depth analysis on the economic impact of the DRG policy. During the process of model development, we comprehensively assessed the implementation effect of the DRG reimbursement system by comparing numerical changes in hospital expenses before and after its implementation while considering differences between the experimental group and control group.

The present study developed the subsequent Difference-in-Differences (DID) mode:

Y_i.t_ = *β*_0_ + *β*_1_ × DRG（Group_i_） + *β*_2_ × Post_t_ + *β*_3_ × PG(Post_t_ × Group_i_) + *β*_4_ × X_i.t_ + *ε*_i.t_

Specifically, Y_it_ represents the natural logarithm of the patient's cost associated with apropriate TCM techniques (ln_tracost_1) and total hospital cost (ln_cost) at time t. The period before January 1, 2021, when the patient is discharged, is considered as the pre-implementation stage of the DRG reimbursement system (post = 0); otherwise, it is categorized as the implementation stage (post = 1). Group_i_ represents the patient's disease group, where group = 0 denotes other disease groups and group = 1 signifies TCM group. PG(Post_t_ × Group_i_) serves as an interaction term used to analyze the impact of DRG reimbursement system implementation on differences between these two disease groups. X_it_ represents a combination of characteristic variables for patients including age, gender, presence of chronic diseases, history of surgery, hospital level, bed number and time-fixed effect control variables. *ε*_it_ random error term.

In the analysis, particular attention is focused on the coefficient *β*3 of the interaction term PG (Post_t_*Group_i_). This coefficient represents the differential impacts of changes in the DRG reimbursement system on expenses related to appropriate techniques of TCM, overall hospital expenses, and surgical expenses across TCM and non-TCM (other) groups. This methodological approach enables an accurate evaluation of the effects of the intervention and elucidates the relationship between the dummy variable and the independent variables in the study (10, 11). The objective of this empirical study is to assess the impact of the DRG reimbursement system on the adoption of appropriate techniques of TCM within TCM hospitals.

#### 2.3.2 The *T*-test in DID analysis

DID analysis is employed to assess the impact of policies, interventions, or treatments on the dependent variable across different groups. It estimates the policy effect by comparing the experimental group (after DRG implementation) with the control group (before the DRG implementation) and calculating their difference. The *T*-test is utilized to examine whether there is a significant average difference between the experimental and control groups. Moreover, it provides a means to evaluate whether the observed difference can be attributed to random variation or if it truly reflects a policy effect. The *T*-test is commonly used in DID analysis as it enables a direct comparison of differences between two groups, aligning with its objective of evaluating whether changes in the dependent variable are associated with policy implementation.

Results of the Difference-in-Differences (DID) model and corresponding visualizations were obtained using Stata 17, while *t*-test analyses were conducted with R version 4.2.3 as the statistical software.

## 3 Result

### 3.1 Descriptive statistical analysis results

#### 3.1.1 The impact of the DRG reimbursement system on three medical cost

**Figure 1:**
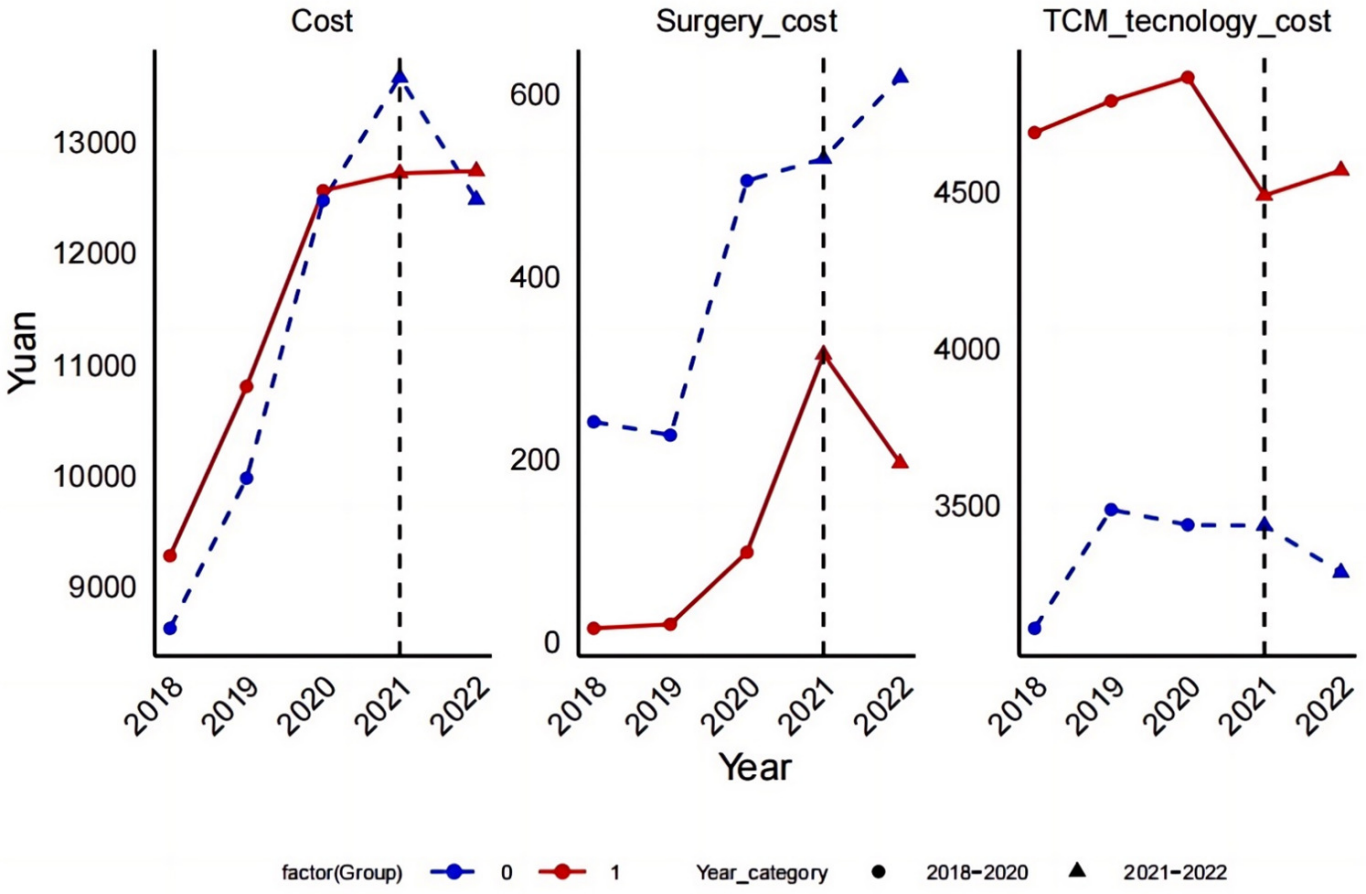
The impact of the DRG payment system implementation on three medical cost items in the experimental and control groups, with a notable cost inflection observed in 2021.

The implementation of the DRG reimbursement system significantly impacted total hospital cost as illustrated in Figure 1. For the TCM group (represented by the red line, group = 1), total cost exhibited a consistent annual increase from 2018, peaking in 2020, and then declining post-policy implementation in 2021. In contrast, the total cost for the other groups (represented by the blue line, group = 0) showed an upward trend from 2018 to 2020, peaking in 2020, followed by a slight rebound in 2022, yet remaining below the previous peak levels. The reduction in overall cost following the implementation of the DRG reimbursement system was less significant compared to the TCM group.

Impact on Surgical cost:for the TCM group, surgical cost peaked both before and after the DRG policy implementation, with a sharp decline following the implementation. In other groups, surgical cost also followed a downward trend after the policy took effect, although the magnitude of the decline was smaller.Impact on appropriate techniques of TCM cost:The treatment cost within the TCM group reached a peak in 2020, and have consistently declined since the implementation of the policy in 2021, continuing this trend into 2022. Conversely, the treatment cost for other groups increased gradually from 2018 to 2020, peaking just before a significant decrease post-policy implementation in 2021. Despite a slight rebound in 2022, the overall trend remains downward.

#### 3.1.2 Analysis of the impact of DRG reimbursement system on medical cost in the two groups

According to Figure 1, it is evident that both groups—TCM and non-TCM(other)—experienced significant fluctuations in their cost before and after the implementation of the DRG reimbursement system. Notably, the surgical cost within the TCM group showed the most significant decrease, suggesting that the DRG system can effectively control the utilization of high-cost medical services. Additionally, the post-DRG implementation period saw a reduction in costs for appropriate techniques of TCM, indicating that DRG may also contribute to reducing cost in specific TCM treatments.

Cost Sensitivity: The cost fluctuations for the TCM group were significantly more pronounced than those observed in the other group, highlighting a heightened sensitivity to the DRG system. This disparity could be attributed to the distinctive nature of TCM treatments or more significant changes in both structure and treatment methodologies within the TCM group under the influence of DRG.

**Figure 2:**
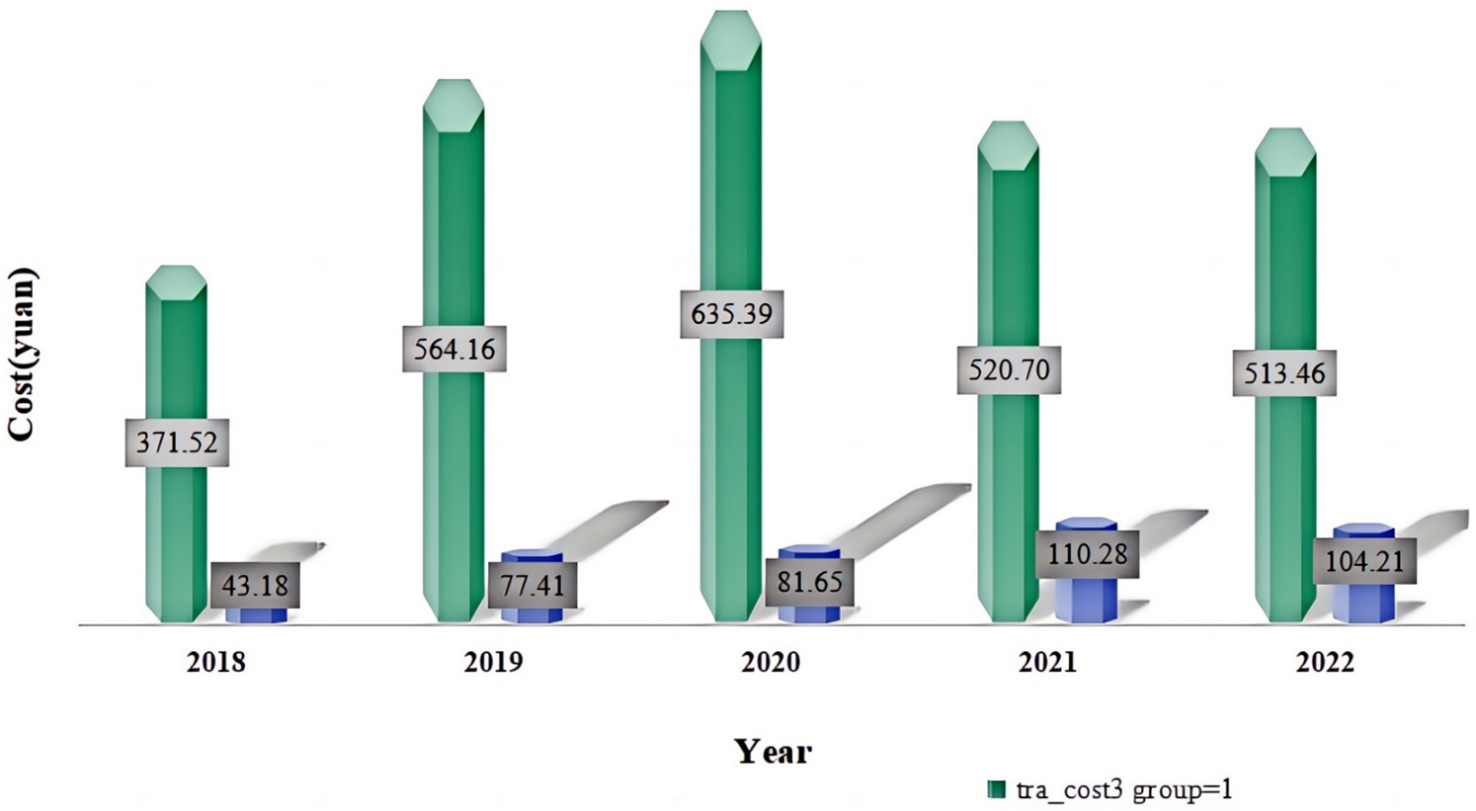
The impact of the DRG payment system implementation on orthopedic technique cost items in the experimental and control groups, with a significant decrease in costs observed in the experimental group starting in 2022. The data indicates a marked reduction in orthopedic technique costs for the experimental group, while costs for the control group remained stable over the same period.Despite an overall upward trend in total cost for both groups, there was a noticeable reduction in cost related to surgery and appropriate techniques of TCM, suggesting that the DRG reimbursement system may optimize resource allocation by minimizing unnecessary medical expenditures. This assertion is corroborated by the observed decrease in orthopedic technique cost(tra_cost3), as depicted in Figure 2, where cost declined from 635.39 Yuan to 520.70 Yuan.

Trend Analysis:Despite an overall upward trend in total cost for both groups, there was a noticeable reduction in cost related to surgery and appropriate techniques of TCM, suggesting that the DRG reimbursement system may optimize resource allocation by minimizing unnecessary medical expenditures. This assertion is corroborated by the observed decrease in orthopedic technique cost, as depicted in Figure 2, where cost declined from 635.39 yuan to 520.70 yuan.

### 3.2 Integrated analysis of DID model and high-dimensional fixed effects linear regression

#### 3.2.1 The impact of the DRG reimbursement system on hospitalization cost

Table 2 presents the statistical analysis results from the regression model on the DRG reimbursement system's effect on healthcare cost. The coefficient *β*_3_ for the interaction term PG (Post*Group) is −0.048, with an associated *p*-value of less than 0.001. This indicates a statistically significant reduction of 4.68% in logarithmic expenses for the experimental group (TCM group) compared to the control group following DRG implementation. Additionally, the coefficient for the time variable (“post”) is 0.017, suggesting a minor positive impact of time on overall expenses. Before DRG implementation, the coefficient for the experimental group was 0.160, indicating that their expenses were relatively higher.

**Table 2:** The impact of DRG policy implementation on the cost of 2 groups.

Variables	Ln(Cost)	Ln(Tcm-tecnology_cost)	Ln(Surgery_cost)
PG(Post^c^Group)	−0.048^a^	−0.154^a^	0.008
	(0.013)	(0.026)	(0.067)
Post	0.017^c^	0.074^a^	0.011
	(0.010)	(0.022)	(0.016)
Group	0.160^a^	1.085^a^	0.088
	(0.021)	(0.046)	(0.133)
Age	0.005^a^	0.002^a^	−0.002^b^
	(0.000)	(0.000)	(0.001)
Gender	−0.041^a^	−0.001	−0.030
	(0.007)	(0.013)	(0.016)
Surgery	0.365^a^	−0.211^a^	1.532^a^
	(0.020)	(0.043)	(0.077)
Chronic disease	0.030^a^	0.030^c^	0.079^a^
	(0.008)	(0.017)	(0.023)
TCM Disease type	0.003^a^	0.007^a^	−0.004
	(0.000)	(0.001)	(0.003)
Constant	8.804^a^	6.407^a^	6.135^a^
	(0.022)	(0.046)	(0.084)
Observations	21,753	21,499	11,224
Adjusted R^2^	0.385	0.582	0.559

Note: data in parentheses are robust standard errors.

The control variables include: age, gender, whether surgery has been performed, presence of chronic diseases, and whether the condition falls under the advantageous disease categories of Traditional Chinese Medicine treatment.

^a^ indicates statistical significance at the 1% level.

^b^ indicates statistical significance at the 5% level.

^c^ indicates statistical significance at the 10% level.

In the specific model analyzing the cost of appropriate techniques of TCM [Ln(Tcm-techniques_cost)], the coefficient for the interaction term PG is −0.154, denoting a significant reduction of 14.27% in cost for the TCM group as a result of the DRG intervention (*p* < 0.001). The “post” variable, serving as an indicator for post-policy implementation, shows a coefficient of 0.074 (*p* < 0.001), indicating a significant overall cost increase post-DRG implementation. Furthermore, the positive coefficient for the group variable at 1.085 (*p* < 0.001) suggests a significant increase in the cost of TCM techniques due to DRG implementation.

Conversely, in the model assessing surgical cost [Ln(Surgery_cost)], the coefficient for the interaction term PG is 0.008 with a *p*-value greater than 0.1, indicating that the implementation of the DRG does not have a statistically significant effect on surgical cost across the groups.In terms of controlling variables, we thoroughly considered the influence of factors such as age, gender, surgical conditions, chronic disease status, and advantage TCM diseases categories on cost. Among these factors, the coefficient of age indicates a slight upward trend in cost as patients' age increases across all aspects. Gender was incorporated into the model as a categorical variable to account for its potential impact on cost. In the Ln(Cost) model, male patients exhibit lower cost; however, in both the Ln(Tcm-tecnology_cost) and Ln(Surgery_cost) models, gender does not significantly affect or has a relatively smaller impact on cost.

The surgical variables are utilized to regulate the impact of surgical factors on cost. In both the Ln(Cost) and Ln(Surgery_cost) models, there was a significant increase in cost for patients who underwent surgery; however, in the variable of Ln(Tcm-techniques_cost), a negative correlation between surgery and cost was observed. Furthermore, chronic disease variables are employed to assess the influence of chronic diseases on cost, and across all models, patients with chronic diseases exhibited higher average cost.

Finally, the TCM disease type was included as a control variable to examine the influence of advantage TCM disease types on cost. In the Ln(Cost) and Ln(Tcm-tecnology_cost) models, the disease type in traditional Chinese medicine exhibited a positive impact on cost; however, this impact was not statistically significant in the of variable of Ln(Surgery_cost).

##### 3.2.1.1 Subgroup heterogeneity analysis

Age-based Subgroup Analysis: Table 2 (refer to Supplementary Material Table S2) reveals distinct impacts of the DRG reimbursement system across different age groups. Notably, hospitalization and surgery costs surged dramatically within the younger cohort (ages 19–45). Conversely, the middle-aged and older groups (ages 46 and above) exhibited more pronounced shifts in surgery and traditional Chinese medicine (TCM) technology costs. For patients aged 60 and above, hospitalization costs remained largely stable; however, the soaring TCM technology and surgery costs highlight the DRG system's profound impact on elderly patients, particularly concerning surgical expenditures.

Chronic Disease-based Subgroup Analysis: Statistical analysis in Table 3 (refer to Supplementary Material Table S3) reveals the DRG reimbursement system significantly impacts hospitalization costs for patients with chronic diseases. Notably, it reduces hospitalization and TCM technology costs, while surgery costs significantly increased.

**Table 3:** *T*-test of the impact of DRG policy implementation on cost.

Year	Group	mean_actual_ln_cost	mean_predicted_ln_cost	mean_difference	percentage_difference(%)	p_value
2018	0	9.3997	9.3466	0.0531	0.5680	0.1132
2018	1	9.0294	9.3827	−0.3533	−3.7665	0.0000
2019	0	9.3735	9.4028	−0.0292	−0.3112	0.1204
2019	1	9.1719	9.3569	−0.1850	−1.9853	0.0000
2020	0	9.4426	9.3904	0.0522	0.5493	0.0000
2020	1	9.3219	9.3543	−0.0324	−0.3521	0.0027
2021	0	9.5230	9.4031	0.1199	1.2663	0.0000
2021	1	9.3472	9.3484	−0.0012	−0.0333	0.8848
2022	0	9.4731	9.3989	0.0742	0.7886	0.0000
2022	1	9.3140	9.3528	−0.0388	−0.4200	0.0001

Note: This table presents the difference between actual costs and predicted costs after the implementation of the DRG policy for surgical procedures, analyzed through a *T*-test. The results show that the *p*-value is less than 0.001, indicating the difference is statistically significant at the 1% level.

#### 3.2.2 Visual analysis of the DID model

**Figure 3:**
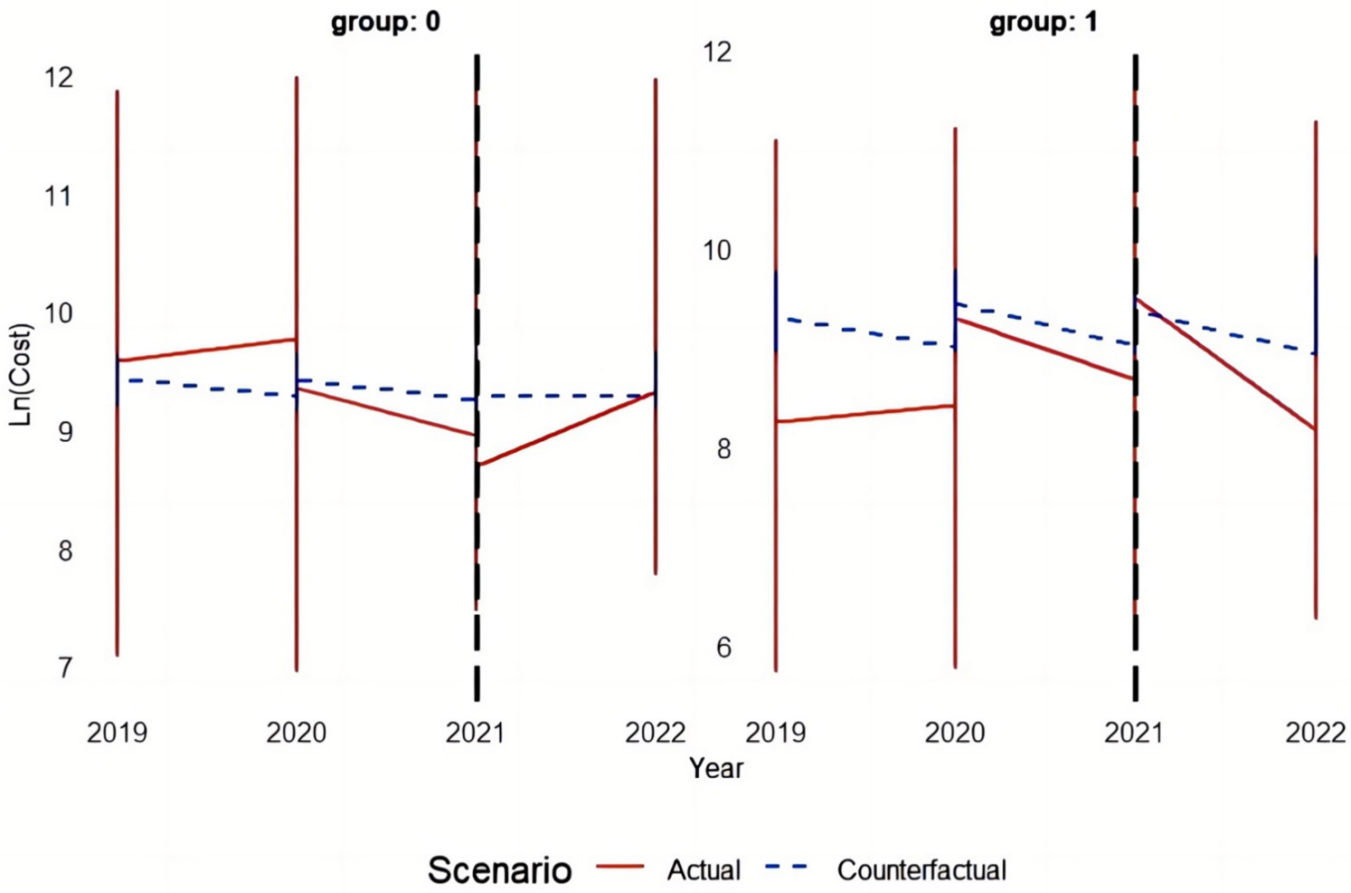
The figure depicts the performance of the TCM group and other groups in terms of both actual and predicted logarithmic total costs from 2019 to 2022.

**Figure 4:**
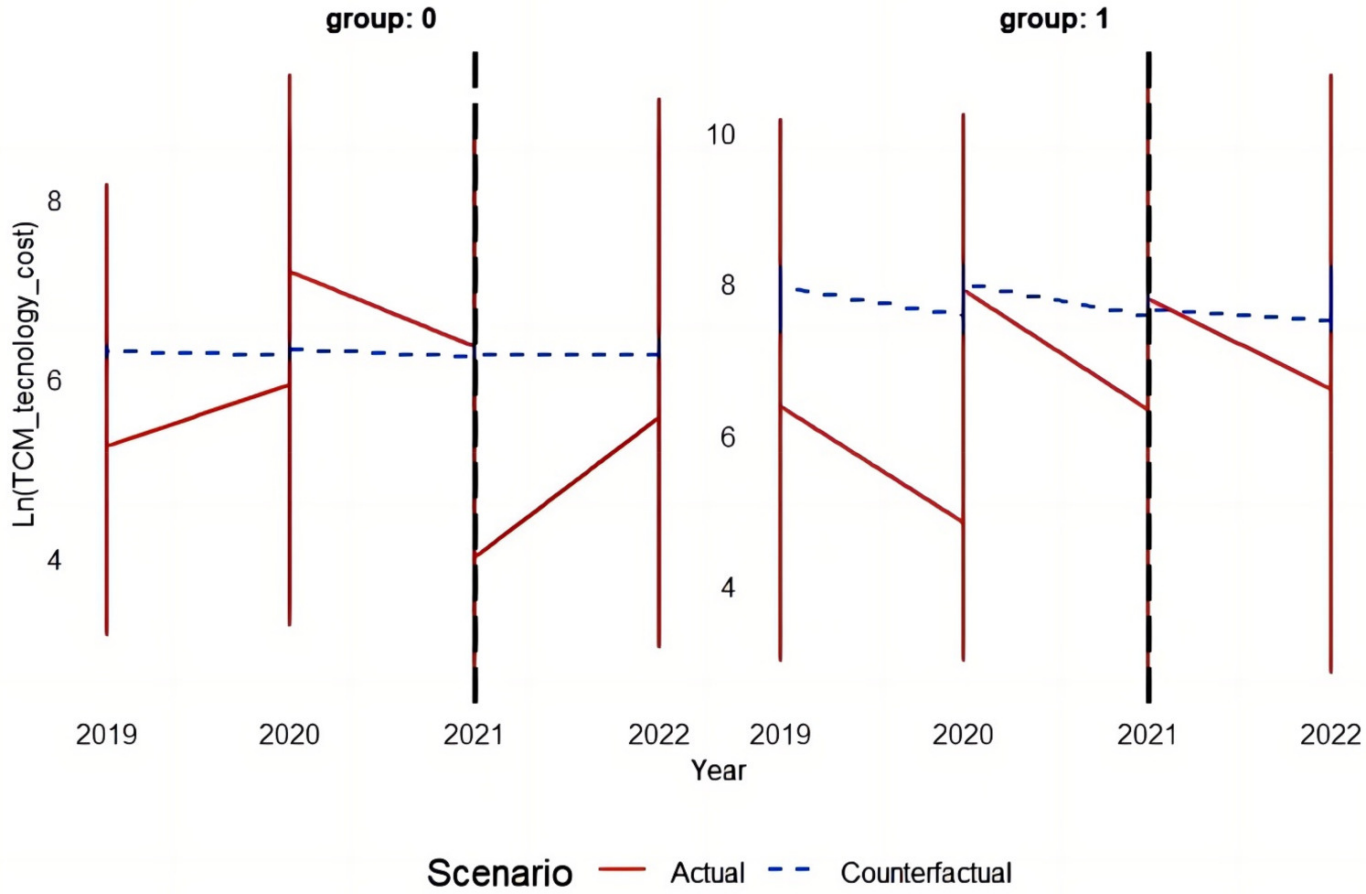
The figure depicts the performance of the TCM group and other groups in terms of both actual and predicted logarithmic TCM costs from 2019 to 2022.

**Figure 5:**
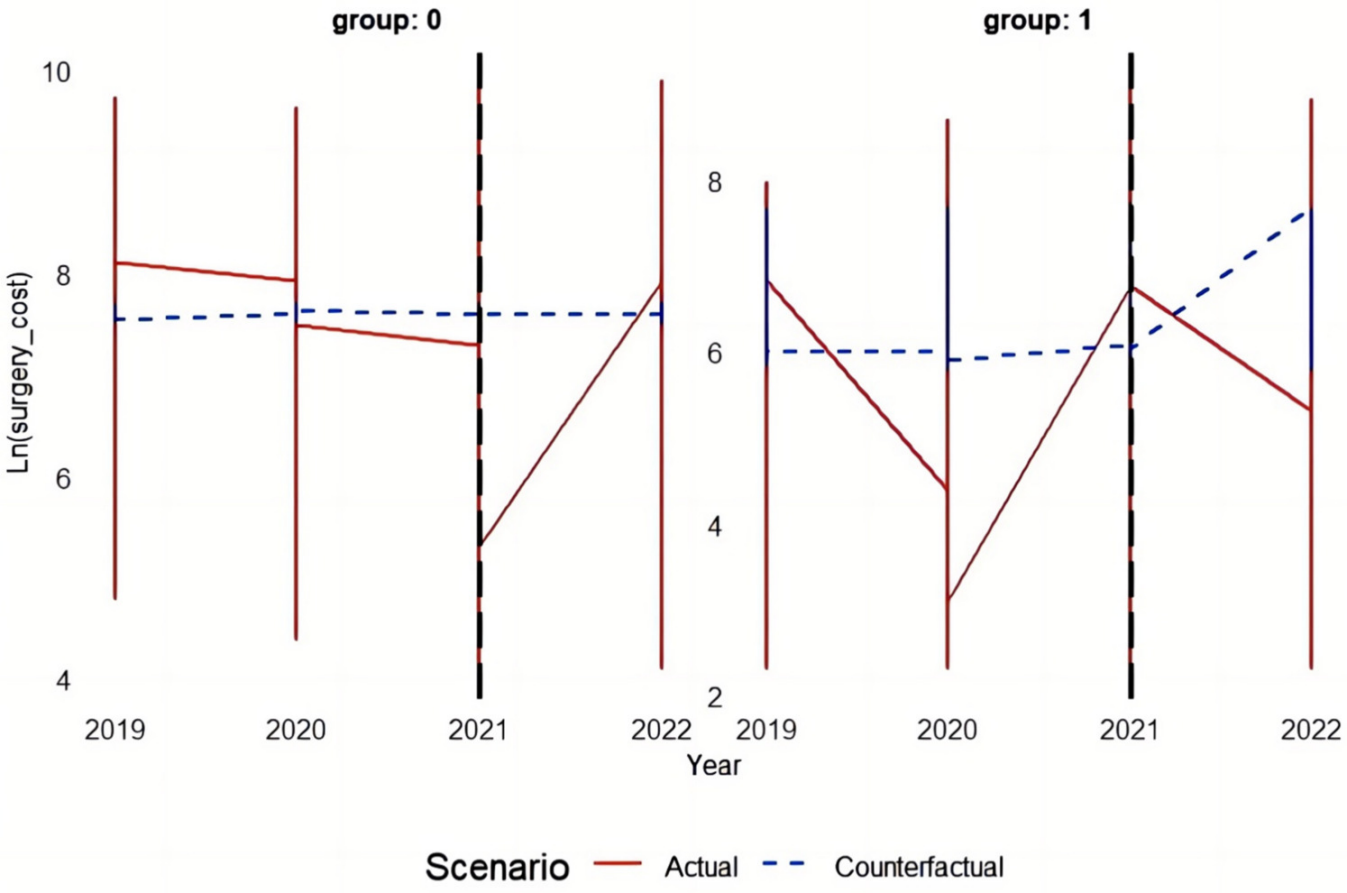
The figure depicts the performance of the TCM group and other groups in terms of both actual and predicted logarithmic surgical costs from 2019 to 2022.

This study employs the Differences-in-Differences (DID) model principle to systematically analyze and validate the effectiveness of the policy by comparing counterfactual results before and after DRG implementation with actual outcomes. Figures 3–5 depict the performance of the TCM group and other groups in terms of both actual and predicted logarithmic expenditures from 2019 to 2022. The red line represents actual expenditures, while the blue dashed line represents predicted expenditures under a counterfactual scenario. Additionally, a vertical black line is used to indicate the commencement year of DRG reimbursement system implementation (2021).

##### 3.2.2.1 The impact of the DRG reimbursement system on total cost

Before the implementation of the policy (in 2019 and 2020), the actual cost in group 0 (other groups) closely aligned with the predicted cost, indicating a consistent approach to cost control prior to DRG implementation. Following the introduction of the DRG in 2021, there was a notable decrease in actual cost compared to predicted cost, which continued into 2022, demonstrating the effective cost management brought about by DRG. As for group 1 (TCM group), there was a slight disparity between actual and predicted cost before policy implementation, suggesting some cost pressure. However, after DRG implementing, there was a significant reduction in actual cost in 2021 that approached predicted levels and further decreased in 2022, highlighting how this policy substantially alleviated the cost burden on the TCM group.

##### 3.2.2.2 The impact of the DRG reimbursement system on the cost of appropriate techniques of TCM

In group 0 (other groups), the difference between the actual cost before and after policy implementation was relatively marginal compared to the predicted cost. However, starting from 2021, the actual cost exhibited a significant reduction in comparison to the predicted cost, indicating successful cost reduction achieved by DRG. In group 1 (TCM group), following DRG implementation, there was a substantial decrease in actual cost as compared to predicted cost, particularly noticeable in 2022, demonstrating a significant impact of the DRG on reducing appropriate TCM technique expenses.

##### 3.2.2.3 The impact of the DRG reimbursement system on surgical cost

In group 0 (other groups), there were relatively minor changes in surgical cost before and after the DRG implementation. However, following a brief surge in actual cost in 2021, a significant downward trend was observed in 2022, potentially indicating cost fluctuations triggered by the DRG implementation. Regarding group 1 (TCM group), the surgical cost exhibited fluctuating characteristics both before and after DRG implementation. Although there was an increase in cost during the DRG implementation period, the actual cost in 2022 surpassed the predicted value, suggesting that the DRG may have had an unintended impact on TCM group's surgical cost.

#### 3.2.3 *T*-test Analysis of cost differences following the implementation of the DRG reimbursement system

##### 3.2.3.1 Impact of DRG reimbursement system implementation on total cost

The *t*-test analysis (see to Table 3) indicates that although the cost difference between the TCM group in 2021 was not statistically significant (*p* = 0.8670), it became significant in 2022 (*p* < 0.05), suggesting a gradual emergence of DRG reimbursement system effects. For the other groups, most years showed no statistically significant differences, thus confirming the accuracy of the prediction model.

##### 3.2.3.2 The impact of the DRG reimbursement system on the cost of appropriate techniques of TCM

The results presented in Table 4 indicate that there was no statistically significant difference in cost between the TCM group and the control group in 2021 (*p* = 0.373). However, this significance increased significantly in 2022 (*p* = 0.00056). In contrast, the difference between the other groups was found to be statistically significant in 2021 (*p* = 1.29e-08), but it decreased in 2022 while still maintaining statistical significance (*p* = 0.00056), thus revealing an unequal impact of the DRG reimbursement system.

**Table 4:** *T*-test of the impact of DRG policy implementation on TCM_tecnology_cost.

Year	Group	mean_actual_ln_TCM_tecnology_cost	mean_predicted_ln_TCM_cost1	mean_difference	percentage_difference(%)	p_value
2018	0	6.4495	6.2650	0.1846	2.9463	0.0100
2018	1	8.2991	8.0333	0.2658	3.3042	0.0000
2019	0	5.9970	6.2844	−0.2874	−4.5647	0.0000
2019	1	8.0367	7.9404	0.0962	1.1109	0.0001
2020	0	6.2697	6.2818	−0.0121	−0.2014	0.5814
2020	1	7.9947	7.9190	0.0758	0.8748	0.0022
2021	0	6.2259	6.3297	−0.1038	−1.6453	0.0000
2021	1	7.8299	7.8455	−0.0156	−0.2261	0.3733
2022	0	6.3921	6.3297	0.0624	0.9845	0.0006
2022	1	7.8808	7.8924	−0.0116	−0.1668	0.5863

Note: This table presents the difference between actual costs and predicted costs after the implementation of the DRG policy for surgical procedures, analyzed through a *T*-test. The results show that the *p*-value is less than 0.001, indicating the difference is statistically significant at the 1% level.

##### 3.2.3.3 The impact of the DRG reimbursement system on surgical cost

The results presented in Table 5 demonstrate that the cost disparity between the TCM group and the control group exhibited statistical significance in 2021 (*p* = 0.0443). Moreover, this disparity became even more pronounced in 2022 (*p* = 0.000), thereby further reinforcing the observability of the DRG reimbursement system effect. Conversely, although the significant difference between the other groups persisted into 2022 with a reduced magnitude (*p* = 0.192), it remained statistically significant (*p* = 1.29E-08) throughout both years, thus indicating both the accuracy of our prediction model and highlighting variations in DRG reimbursement system effects.

**Table 5:** *T*-test of the impact of DRG policy implementation on surgery_cost.

Year	Group	mean_actual_ln_sur_cost	mean_predicted_ln_sur_cost	mean_difference	percentage_difference(%)	p_value
2018	0	7.6628	7.5899	0.0729	0.9618	0.0667
2018	1	6.1565	6.1764	−0.0199	−0.3875	0.9736
2019	0	7.4873	7.5835	−0.0961	−1.2710	0.0000
2019	1	4.8154	6.0293	−1.2139	−20.2845	0.0000
2020	0	7.5692	7.5872	−0.0181	−0.2418	0.2612
2020	1	5.9421	6.1973	−0.2553	−4.4236	0.0473
2021	0	7.6892	7.5973	0.0919	1.2032	0.0000
2021	1	6.8974	6.7676	0.1298	1.6847	0.0443
2022	0	7.6106	7.5934	0.0172	0.2220	0.1917
2022	1	6.1353	6.4931	−0.3578	−6.1184	0.0000

Note: This table presents the difference between actual costs and predicted costs after the implementation of the DRG policy for surgical procedures, analyzed through a *T*-test. The results show that the *p*-value is less than 0.001, indicating the difference is statistically significant at the 1% level.

#### 3.2.4 Robustness check: impact of DRG reimbursement system after One year

To evaluate the robustness of our findings, we performed an additional sensitivity analysis, shortening the policy implementation period to one year post-policy instead of the two-year time frame used in the primary DID analysis. Crucially, the results under this condensed one-year period align consistently with those from the two-year window, demonstrating the stability of our conclusions across varying policy timelines. Table 4 (refer to Supplementary Material Table S4) details the DID results for the one-year post-policy period:Ln(Cost):The Post*Group interaction coefficient is −0.0879593, statistically significant at the 1% level. This indicates the DRG payment system significantly reduced hospitalization costs for the treatment group, mirroring the two-year period findings.Ln(Tcm-technology_cost):The Post*Group coefficient is −0.1261736, also significant at the 1% level. This suggests the DRG system significantly lowered traditional Chinese medicine (TCM) technology costs within the treatment group, consistent with the two-year results. Ln(Surgery_cost): The PostGroup interaction term coefficient is 0.1590512, significant at the 5% level, indicating the DRG payment system led to a significant increase in surgery costs for the treatment group, again matching the findings under the longer implementation period.

Furthermore, the control variables (Age, Gender, Surgery, Chronic disease, and TCM Disease type) exhibit consistency across all models, further reinforcing the robustness of the results. The adjusted R-squared values—0.3896 for hospitalization costs, 0.5907 for TCM technology costs, and 0.5444 for surgery costs—suggest a strong explanatory power and good model fit. Collectively, these results, derived from a one-year post-policy implementation period, powerfully reaffirm the consistency and reliability of our core findings, underscoring the robustness of the DRG payment system's impact on hospitalization, TCM technology, and surgery costs.

The economic impact of the DRG reimbursement system was comprehensively analyzed in this study using the double difference method (DID), visualization, and *t*-test. The DID analysis revealed a significant reduction of cost in the TCM group, particularly in appropriate techniques of TCM, after the implementation of the DRG policy. There was a notable interaction effect between the DRG policy and group, indicating an effective 14.27% decrease in technical cost for the TCM group. The visualization clearly depicted cost changes from 2019 to 2022 between the TCM group and other groups, highlighting a substantial cost reduction following DRG policy implementation. Furthermore, *t*-test results confirmed this trend by demonstrating a significant increase in cost differences between the TCM group and other groups in 2022 while consistently validating the accuracy of our predictive model for other groups' cost. These analyses collectively provide evidence for different cost areas and groups regarding the impact of DRG reimbursement system, with particular emphasis on its success in controlling TCM technical cost. In addition to the observed cost reductions, the timing of these effects warrants further consideration.

This delayed effect of the DRG policy, becoming statistically significant only in 2022, suggests that the full impact of the policy was not immediately evident. This delayed impact can be more reasonably interpreted as a result of institutional learning. Healthcare providers and institutions likely needed time to adjust to the new DRG system, learn from initial implementation, and adapt their practices accordingly. Over time, as they gained a better understanding of the policy's structure and incentives, the policy's effects became more pronounced. This process of gradual adaptation, or “institutional learning,” explains the delayed emergence of significant statistical effects, and it emphasizes the importance of time for full compliance and adjustment to new reimbursement frameworks.

### 3.3 The impact of DRG reimbursement system on the cost of different categories of appropriate techniques of TCM

Based on the DID model, we conducted an analysis of six categories of the appropriate techniques of TCM. The findings indicate that the implementation of the DRG reimbursement system has had a significant economic impact on the cost associated with various the appropriate techniques of TCM. Tables 6, 7 reveals that since the adoption of the DRG reimbursement system, there has been a decrease in total cost for Chinese medicine external treatment techniques by −0.049 units or 4.78%. Similarly, acupuncture techniques has experienced a reduction in total cost by −0.084 units or 8.06%, which is statistically significant (*p* < 0.001). Furthermore, all categories of the appropriate techniques of TCM have witnessed substantial decreases in cost: Chinese medicine external treatment techniques decreased by −0.147 units or 13.67% (*p* < 0.001), Chinese medicine orthopedic techniques decreased by −0.154 units or 14.27% (*p* < 0.001), acupuncture techniques decreased by −0.184 units or 16.% (*p* < 0.001), and Chinese medicine special therapy decreased by −0.098 units or %9.3 (*p* < .05). These reductions are all statistically significant.

**Table 6:** The impact of DRG reimbursement system implementation on the total cost of using various TCM appropriate techniques.

TCM tecnology name	TCM external treatment	TCM orthopedic techniques	TCM acupuncture techniques	TCM massage techniques	TCM colorectal techniques	TCM special therapies
Variables	Ln(Cost)					
PG(Post^c^Group)	−0.049^a^	−0.018	−0.084^a^	−0.017	0.007	−0.029
	(0.013)	(0.024)	(0.018)	(0.056)	(0.054)	(0.022)
Control variables:surgery	0.370^a^	0.279^a^	0.301^a^	0.041	−0.087	0.342^a^
	(0.020)	(0.029)	(0.025)	(0.134)	(0.063)	(0.035)
Control variables:chronic diesase	0.028	0.018	0.022	0.016	0.023	0.009
	(0.009)	(0.014)	(0.010)	(0.022)	(0.048)	(0.013)
Observations	20,352	6,817	13,472	2,629	1,012	7,950
Adjusted R^2^	0.400	0.407	0.355	0.430	0.326	0.424

Note: Data in parentheses are robust standard errors.

The control variables include: age, gender, whether surgery has been performed, presence of chronic diseases, and whether the condition falls under the advantageous disease categories of Traditional Chinese Medicine treatment.

^a^ indicates statistical significance at the 1% level.

^b^ indicates statistical significance at the 5% level.

^c^ indicates statistical significance at the 10% level.

**Table 7:** The impact of DRG reimbursement system implementation on the cost of using various TCM appropriate techniques.

TCM tecnology name	TCM external treatment	TCM orthopedic techniques	TCM acupuncture techniques	TCM massage techniques	TCM colorectal techniques	TCM special therapies
Variables	Ln(TCM_tecnology_cost)					
PG(Post^c^Group)	−0.147^a^	−0.154^a^	−0.184^a^	0.100	0.030	−0.098^b^
	(0.026)	(0.038)	(0.034)	(0.092)	(0.096)	(0.035)
Control variables: surgery	−0.196^a^	−0.280^a^	−0.308^a^	−0.251^c^	−0.337^a^	−0.286^a^
	(0.043)	(0.048)	(0.050)	(0.146)	(0.097)	(0.064)
Control variables: chronic diesase	0.022	0.039	0.021	−0.005	0.016	0.004
	(0.017)	(0.020)	(0.018)	(0.029)	(0.082)	(0.021)
Observations	20,352	6,817	13,472	2,629	1,012	7,950
Adjusted R^2^	0.586	0.656	0.584	0.694	0.520	0.603

Note: Data in parentheses are robust standard errors.

The control variables include: age, gender, whether surgery has been performed, presence of chronic diseases, and whether the condition falls under the advantageous disease categories of Traditional Chinese Medicine treatment.

^a^ indicates statistical significance at the 1% level.

^b^ indicates statistical significance at the 5% level.

^c^ indicates statistical significance at the 10% level.

Additionally, our analysis revealed that surgical patients within the group utilizing Chinese medicine techniques exhibited significantly lower cost associated with these treatments; however, their overall expenses were generally higher due to increased surgical cost and other related expenditures.This finding suggests a decline in utilization of the appropriate techniques of TCM among surgical patients but an increase in total cost attributed to surgery-related expenses and other factors.This discovery provides valuable insights for comprehensively understanding and optimizing the application of DRG reimbursement systems within the field of TCM.

### 3.4 The impact of DRG reimbursement system on the cost of different departments of appropriate techniques of TCM

A comprehensive analysis was conducted on the eight departments most impacted by the DRG reimbursement system, namely acupuncture, cardiology, tuina, pain management, geriatrics, stomach and spleen disorders, anal and rectal conditions, and orthopedics (see Tables 8, 9). The following conclusions were derived: Following the implementation of the DRG reimbursement system, there were varying trends in total cost for disease groups utilizing appropriate techniques of TCM across different departments. As shown in Tables 8, 9, significant reductions of 0.209 units (18.86%, *p* < 0.001) were observed in total cost within the cardiology department; conversely, notable increases of 0.112 units (11.85%, *p* < 0.001) occurred in total cost within the acupuncture department; additionally, substantial increases of 0.522 units (68.54%, *p* < 0.05) were noted in total cost within the pain management department.

**Table 8:** The impact of DRG reimbursement system on departmental total expenses.

TCM_tecnology_name	Orthopedics	Anorectal department	Spleen and stomach diseases	Geriatric	Pain treatment	Massage	Cardiovascular internal medicine	Acupuncture
Variables	Ln(Cost)							
PG(Post^c^Group	−0.140^c^	−0.005	0.003	−0.102	0.522^b^	0.344	−0.209^a^	0.112^a^
	(0.077)	(0.037)	(0.054)	(0.100)	(0.230)	(0.448)	(0.053)	(0.026)
Control variables:surgery	0.174	−0.019	−0.119^b^	−0.108	0.373^c^	0.263	−0.072	0.147
	(0.141)	(0.045)	(0.057)	(0.136)	(0.217)	(0.215)	(0.169)	(0.114)
Control variables: chronic diesase	0.031	0.011	−0.061^b^	−0.061^b^	0.016	−0.001	0.090^a^	0.043
	(0.046)	(0.036)	(0.021)	(0.030)	(0.036)	(0.031)	(0.026)	(0.018)
Observations	1,020	1,773	2,576	1,292	690	1,060	1,160	3,883
Adjusted R^2^	0.592	0.321	0.326	0.257	0.318	0.190	0.533	0.109

Note: Data in parentheses are robust standard errors.

The control variables include: age, gender, whether surgery has been performed, presence of chronic diseases, and whether the condition falls under the advantageous disease categories of Traditional Chinese Medicine treatment.

^a^ indicates statistical significance at the 1% level.

^b^ indicates statistical significance at the 5% level.

^c^ indicates statistical significance at the 10% level.

**Table 9:** Impact of DRG reimbursement system implementation on the cost of Chinese medicine appropriate techniques in departments.

TCM_tecnology_name	Orthopedics	Anorectal department	Spleen and stomach diseases	Geriatric	Pain treatment	Massage	Cardiovascular internal medicine	Acupuncture
Variables	Ln(TCM_tecnology_cost)							
PG(Post^c^Group)	−0.294^b^	0.074	0.383^b^	−0.399	0.435	0.956	−0.151	0.174^a^
	(0.125)	(0.068)	(0.132)	(0.259)	(0.383)	(0.759)	(0.098)	(0.030)
Control variables:surgery	−2.013^a^	−0.281^a^	−0.679^b^	−0.937^a^	−0.396	−0.968	−0.343	−0.091
	(0.168)	(0.065)	(0.274)	(0.173)	(0.404)	(0.714)	(0.342)	(0.250)
Control variables:chronic diesase	−0.021	−0.053	0.022	−0.086	0.039	0.022	0.096^c^	0.058
	(0.077)	(0.067)	(0.066)	(0.063)	(0.094)	(0.040)	(0.050)	(0.021)
Observations	981	1,768	2,451	1,287	690	1,059	1,145	3,883
Adjusted R^2^	0.608	0.486	0.0834	0.285	0.0880	0.225	0.348	0.0984

Note: Data in parentheses are robust standard errors.

The control variables include: age, gender, whether surgery has been performed, presence of chronic diseases, and whether the condition falls under the advantageous disease categories of Traditional Chinese Medicine treatment.

^a^ indicates statistical significance at the 1% level.

^b^ indicates statistical significance at the 5% level.

^c^ indicates statistical significance at the 10% level.

In terms of changes in the cost of appropriate techniques of TCM, the cost of orthopedics has significantly decreased by 0.294 units, equivalent to a reduction of 30.3% (*p* < 0.001). Conversely, the cost of acupuncture and stomach and spleen medicine has experienced a significant increase by 0.174 units, corresponding to an increment of 18.88% (*p* < 0.001) and 0.383 units, representing a rise of 46.67% (*p* < 0.001), respectively.

The implementation of surgical interventions has resulted in varying economic impacts across different medical departments. Notably, in the cardiology department, both surgical interventions and cost for appropriate techniques of TCM have exhibited a simultaneous upward trend. This increase may be attributed to the unique disease characteristics and the specific treatment approaches required in cardiology. Conversely, in departments such as geriatrics, gastroenterology (specifically spleen and stomach disorders), proctology (anus and rectum conditions), and orthopedics, the introduction of surgical procedures has led to a decrease in expenses associated with appropriate techniques of TCM.

This trend suggests a potential substitutional or complementary relationship between surgical interventions and TCM techniques, with a particularly noteworthy observation in the orthopedics department.Herein, the frequent recourse to Western medical surgeries has resulted in a marked decline in the expenditure pertaining to orthopedic procedures of TCM. This diminution in cost is attributable to the superior efficiency demonstrated by Western surgical interventions under the DRG reimbursement system, which subsequently lessens the necessity for orthopedic techniques of TCM. Such observations lend further credence to the presumption of an interchangeable or supplementary nexus existing between surgical procedures and pertinent appropriate techniques of TCM.

## 4 Discussion and conclusion

The implementation of the DRG reimbursement system has sparked profound transformations across the healthcare landscape, especially impacting its interaction with Traditional Chinese Medicine (TCM). This analysis centers on four critical dimensions emerging from the evidence: (1) the glaring gaps within DRG design for TCM, (2) the observable cost adaptations triggered by DRG, (3) the spectrum of institutional responses to the DRG framework, and (4) the looming long-term risks alongside viable policy options.

### 4.1 DRG reform and Its impact on healthcare system: clinical and institutional adjustments

The implementation of the DRG (Diagnosis-Related Group) reimbursement system marks a significant starting point, initiating changes within the healthcare system. This reform drives both clinical and institutional-level transformations, with important implications for cost control, treatment effectiveness, and financial management(refer to Supplementary Figure S2).

#### 4.1.1 Clinical-level adjustments

(1) Treatment Adjustments: Healthcare institutions adjust treatment plans in response to the DRG payment standards. This may involve reducing the use of high-cost treatments or adopting more efficient, cost-effective methods. The DRG system's financial incentives encourage providers to prioritize treatments that align with the reimbursement structure, thus affecting the selection and intensity of medical interventions.
(2) Patient Outcomes: As treatment plans are modified in line with DRG guidelines, these changes impact patient outcomes. By aligning clinical practices with the DRG system, healthcare providers aim to improve the effectiveness and efficiency of care, potentially enhancing patient recovery and reducing unnecessary medical interventions.

#### 4.1.2 Institutional-level adjustments

(1) Financial Adjustments: Under the DRG system, healthcare institutions adjust their financial management practices to optimize costs. This includes reducing expenditures on treatments that do not meet DRG standards or that are not sufficiently reimbursed, thereby improving overall financial sustainability. Institutions may also implement cost-control strategies to align with the reimbursement structure, ensuring that the financial burden remains within acceptable limits.
(2) Healthcare Resource Allocation: Hospitals are required to adapt their resource allocation to accommodate the DRG payment policies. This may involve re-evaluating how resources such as staff, equipment, and facilities are distributed across departments. By improving efficiency and optimizing resource distribution, institutions can better align with the DRG framework and ensure a more cost-effective delivery of healthcare services.
(3) Policy Adaptation and Institutional Learning: Over time, healthcare providers gradually adapt to the DRG system, learning to navigate the new reimbursement rules. This process, referred to as institutional learning, enables hospitals to improve their compliance with DRG standards, thereby enhancing their efficiency and optimizing resource use. As providers gain more experience, they can refine their strategies to better meet the financial goals of the DRG system without compromising the quality of care.

#### 4.1.3 Cost and economic outcomes

(1) Cost Control: Through both policy and clinical adjustments, the DRG system contributes to the control of medical costs. By incentivizing healthcare providers to optimize their treatment approaches, the system effectively reduces unnecessary or inefficient healthcare expenditures, fostering a more sustainable healthcare environment.
(2) Economic Impact: The ultimate economic effect of the DRG system includes its influence on hospital financial health and patient cost burden. As institutions adjust to the new payment structure, they are better able to manage costs, which can lead to improved financial stability for hospitals and reduced out-of-pocket expenses for patients. This creates a more economically efficient healthcare model, benefiting both providers and recipients.

In conclusion, the DRG system serves as a catalyst for reforming healthcare practices, prompting both clinical and institutional-level changes that contribute to improved cost control and economic outcomes. The ongoing adaptation to these new systems and policies underscores the importance of institutional learning in achieving long-term success under DRG reimbursement frameworks.

### 4.2 DRG design gaps with TCM

The DRG reimbursement system, rooted in Western medicine principles, optimizes cost-efficiency by categorizing diseases into standardized groups with predetermined payment rates. However, its design fails to adequately accommodate the unique characteristics of Traditional Chinese Medicine (TCM), creating significant challenges for integrating TCM effectively within the DRG framework.

Traditional Chinese Medicine (TCM) adheres to a profoundly unique diagnostic and treatment philosophy compared to the standardized, evidence-based protocols characteristic of Western medicine. TCM diagnoses pivot on “syndrome differentiation,” embracing a holistic, individualized treatment approach rather than targeting isolated diseases or symptoms. Conversely, the DRG system operates through precisely defined diagnostic codes and strictly standardized treatment pathways typical of Western medicine. This fundamental divergence creates an inherent design gap: the DRG framework, structured around discrete disease entities, is fundamentally at odds with and systematically excludes many core TCM techniques—particularly those vital for prevention, health maintenance, and managing complex syndromes.

Furthermore, the exclusion of TCM techniques from DRG groupings significantly widens this disparity. Vital components of TCM treatment plans—such as acupuncture, herbal medicine, and Tuina therapy—are frequently omitted from the DRG system's reimbursement structures. The system's overwhelming focus on high-volume, high-cost treatments, typically involving surgical and pharmaceutical procedures, offers scant incentive for hospitals to prioritize or innovate within TCM. Consequently, TCM hospitals struggle to secure adequate reimbursement for their services, undermining their financial viability and ability to operate sustainably under the DRG framework.

### 4.3 Observed cost adaptations under DRG

The DRG reimbursement system has a powerful impact on the way healthcare providers manage costs, incentivizing them to optimize the utilization of medical services. This economic motivation leads to observable shifts in service delivery patterns, particularly in the context of Traditional Chinese Medicine (TCM). Our rigorous statistical analysis reveals that following the implementation of the DRG system, hospitals specializing in TCM experienced a significant decline in both total costs and expenditures allocated to appropriate techniques of TCM. These reductions were most pronounced in areas where treatments were either excluded from DRG reimbursement or burdened with low reimbursement rates.

High-cost TCM treatments—such as Chinese herbal decoctions, specialized non-drug therapies, and complex acupuncture procedures—saw notable decreases in utilization as hospitals sought to minimize expenses. Healthcare providers responded by adopting more cost-effective treatment options that better align with the DRG system's reimbursement structures. This shift toward lower-cost, less complex treatments mirrors the behavior observed globally under DRG systems. Essentially, the DRG payment structures unintentionally restrict the use of TCM methods that are perceived as less cost-efficient or excluded from groupings, which may erode the holistic care that TCM uniquely offers.

The observed shift in cost management extends beyond TCM to other treatment modalities. While surgical costs remained relatively stable, the declining use of TCM techniques casts doubt on its enduring value and long-term sustainability within the healthcare system. In orthopedics, an interesting and significant trend has emerged: the frequent utilization of Western medical surgeries has led to a noticeable reduction in the costs associated with TCM orthopedic techniques. This phenomenon is closely related to the incentives embedded in the DRG reimbursement system, which influences both the selection of treatment methods by healthcare providers and the cost dynamics of these methods.

(1) Higher Reimbursement Rates for Western Surgery in Orthopedics

One of the key factors contributing to the observed cost reduction in TCM orthopedic techniques is the higher reimbursement rates associated with Western surgical procedures in the DRG system. Under the DRG framework, medical treatments are assigned specific weights, which determine the reimbursement amount. These weights are based on a variety of factors, including the perceived complexity, resource utilization, and cost of the treatment.

In orthopedics, Western surgical procedures such as joint replacements, spinal surgeries, and fracture repairs are assigned higher DRG weights due to their complexity, higher resource usage, and significant impact on patient outcomes. As a result, these surgeries are reimbursed at a higher rate compared to TCM techniques. Consequently, healthcare providers, particularly those in orthopedic specialties, are incentivized to prioritize Western surgical interventions due to the higher financial returns associated with these procedures.

(2) Lower Reimbursement for TCM Orthopedic Techniques

In contrast, traditional Chinese medicine (TCM) orthopedic techniques, such as acupuncture, tuina (Chinese therapeutic massage), and herbal treatments, are generally not included in the DRG payment groups or are assigned lower reimbursement rates. This results in TCM orthopedic techniques being perceived as less financially attractive within the DRG system, especially when compared to the more profitable Western surgical options. Because TCM techniques are not adequately compensated under the DRG system, hospitals and healthcare providers may reduce their utilization of these methods in favor of procedures with higher reimbursement rates. The low reimbursement weight assigned to TCM orthopedic techniques by the DRG system thus leads to a decrease in demand for these treatments, as hospitals aim to optimize their revenue by focusing on treatments that yield higher payments. This aligns with economic theories related to financial incentives, where healthcare providers adjust their practices to maximize reimbursement, often at the expense of non-reimbursed or under-reimbursed services.

(3) Substitution or Complementarity Between Surgery and TCM

This dynamic between Western surgery and TCM techniques in orthopedics suggests a potential substitution effect, where providers choose to adopt Western surgical procedures instead of TCM techniques due to the higher financial incentives. In areas where DRG reimbursement for surgery is higher, providers may increasingly favor surgery, reducing the role of TCM in orthopedic care.However, it is also important to note that the relationship between surgery and TCM is not strictly one of substitution. In certain cases, TCM techniques may complement Western surgeries, enhancing patient outcomes. For example, TCM therapies such as acupuncture and tuina may be used to alleviate pain, improve rehabilitation, and promote healing post-surgery. In this sense, TCM techniques can serve as complementary treatments that enhance the overall effectiveness of surgical interventions, providing patients with a more holistic approach to care.

Thus, while the DRG system may encourage substitution in some cases, particularly where the reimbursement for surgery is substantially higher, there are instances where TCM techniques and Western surgery can work synergistically, particularly in post-surgical rehabilitation and pain management.

### 4.4 Institutional responses to the DRG system

Healthcare institutions, particularly TCM hospitals, have strategically responded to the DRG reimbursement system, adapting their service offerings to align with these new payment incentives. This institutional adaptation stems from the dual imperative to comply with the DRG system's financial constraints while simultaneously striving to maintain their patient base and uphold medical outcomes.

A pivotal response involves significantly reducing the utilization of non-reimbursed or low-reimbursement TCM techniques. TCM hospitals, driven by the urgent need to curb costs, have increasingly embraced more cost-effective practices. This often translates into substituting traditional TCM techniques with reimbursable Western medical treatments under the DRG framework. This substitution effect manifests clearly: surgical procedures with higher reimbursement rates see increased use, while TCM techniques failing to meet DRG criteria experience a corresponding decline (26, 35, 39, 40).

Furthermore, TCM hospitals have implemented operational adjustments, strategically focusing on treatments with stronger DRG reimbursement potential. These adjustments frequently include expanding services that integrate TCM with Western medicine, particularly within the outpatient sector where reimbursement opportunities for pure TCM treatments remain constrained. By deliberately shifting focus towards outpatient services, hospitals aim to bolster financial viability while still providing an integrated form of care that incorporates TCM elements (33, 34).

However, these institutional responses carry significant potential drawbacks. Curtailing the use of traditional TCM techniques while favoring more cost-effective treatments risks eroding TCM's distinctive advantages—especially its holistic approach to patient care. Over time, such strategic pivots could jeopardize the fundamental value proposition of TCM, weakening its role within integrated healthcare and diminishing the cherished cultural and medical legacy embodied in these practices.

### 4.5 Long-term risks and policy options

While the DRG system has successfully reduced healthcare costs in the short term, it presents several long-term risks that could undermine the future of TCM and its integration with Western medicine. One significant danger is the gradual erosion of TCM's core capabilities, particularly in vital areas such as preventive care, chronic disease management, and individualized treatment planning. The declining utilization of specialized TCM techniques under DRG could jeopardize the transmission of invaluable traditional knowledge and skills, potentially leading to a deterioration in TCM service quality and an increasingly diminished role within the broader healthcare landscape.

Moreover, the substitution between surgery and TCM poses significant challenges in balancing the use of both systems. While surgery and TCM may complement each other in some areas, such as cardiology, they often act as substitutes in fields like orthopedics. The increasing reliance on Western medicine surgeries, coupled with reduced use of TCM, threatens to disrupt the delicate balance between these two approaches, potentially undermining the holistic treatment model uniquely offered by TCM.

To address these long-term risks, it is imperative to reconsider the DRG system's design. Policy reforms must prioritize integrating TCM into the DRG framework in a manner that fully recognizes its distinctiveness and ensures fair reimbursement for these services. Expanding the DRG reimbursement system to encompass a broader range of TCM techniques, particularly in outpatient services, promises to deliver a more comprehensive approach to patient care and safeguard TCM's relevance within the healthcare system (39, 40).

Furthermore, a truly balanced healthcare policy must emphasize the profound importance of preventive medicine—a cornerstone strength of TCM. By weaving TCM's preventative strategies into the DRG framework, policy makers can significantly enhance healthcare cost-effectiveness while ensuring TCM continues its vital role in safeguarding public health and curbing long-term healthcare expenditures.

## 5 Conclusion and policy recommendation

This study examines the effects of DRG reimbursement on TCM technique usage and disparities across techniques and departments. Using DID methodology, it analyzes 2018–2022 cost data from three Chinese medicine hospitals in W City, China. Results show a significant 4.68% decrease in total TCM costs after DRG implementation (*p* < 0.001), with a 14.27% reduction in specific TCM techniques (*p* < 0.001), but no significant changes in surgical costs. These findings suggest that Chinese medicine hospitals have adjusted their use of TCM techniques to balance quality with DRG medical insurance policies. Policymakers should carefully consider the alignment between DRG policies and TCM treatment methods to promote fairness and efficiency while preserving TCM advantages.

Based on the diverse implementation of medical insurance payment methods across regions and practical TCM hospital payment experience, Table 5 (refer to Supplementary Material Table S5) presents the major current TCM-DRG payment approaches. These schemes offer multiple alternative pathways, each possessing distinct strengths and limitations in integrating traditional Chinese medicine within the DRG framework. Given varying regional healthcare needs and policy environments, the feasibility and effectiveness of each approach will differ significantly. The lump-sum payment plus supplementary payment model merges fixed payments with supplementary compensation tied to TCM therapeutic outcomes. This strategy effectively controls overall costs while simultaneously motivating hospitals to deliver high-quality TCM care through performance-based incentives. Offering considerable flexibility, this model is well-suited for long-term adoption, though it carries a potential risk of under-addressing complex medical conditions.

Therefore, policymakers should evaluate the implementation effects of each scheme according to the characteristics of their local healthcare systems. They should also make reasonable selections and adjustments by incorporating regional pilot experiences and practical conditions to ensure that traditional Chinese medicine can fulfill its unique role within modern healthcare systems. In conclusion, while the DRG reimbursement system remains a practical approach to controlling healthcare costs and improving efficiency, the integration of TCM requires careful adaptation of the payment structures. A well-designed payment model that incorporates the unique characteristics of TCM will help ensure that these services are adequately valued, leading to better care outcomes and promoting the continued development of TCM in the healthcare system.

## Data Availability

The datasets presented in this article are not readily available because due to the fact that this research data is individual medical insurance reimbursement data from the project team, it cannot be provided to others for use. Requests to access the datasets should be directed to tiantiantt870103@163.com.
