# Supplementary figures and images for: The impact of DRG reimbursement system on appropriate techniques of traditional Chinese medicine—evidence from pilot cities with traditional Chinese medicine hospitals in China
Source: Front Health Serv. 2025 Aug 4;5:1441482. doi: 10.3389/frhs.2025.1441482 (PMC12358404; doi:10.3389/frhs.2025.1441482)

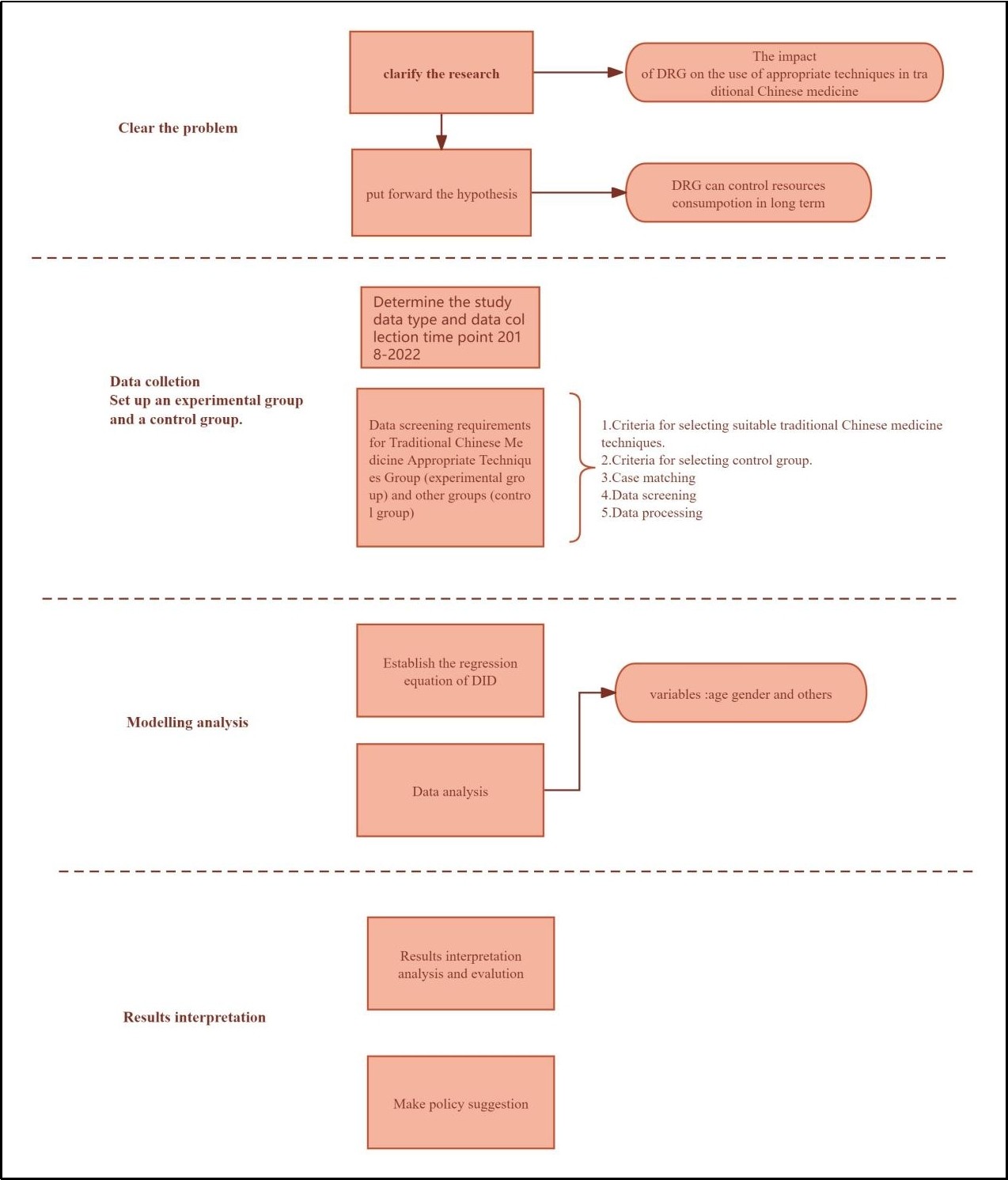

Supplement: Supplementary file 1 [file Datasheet1.zip › Supplementary/Supplementary-FIGURE 1.jpg]

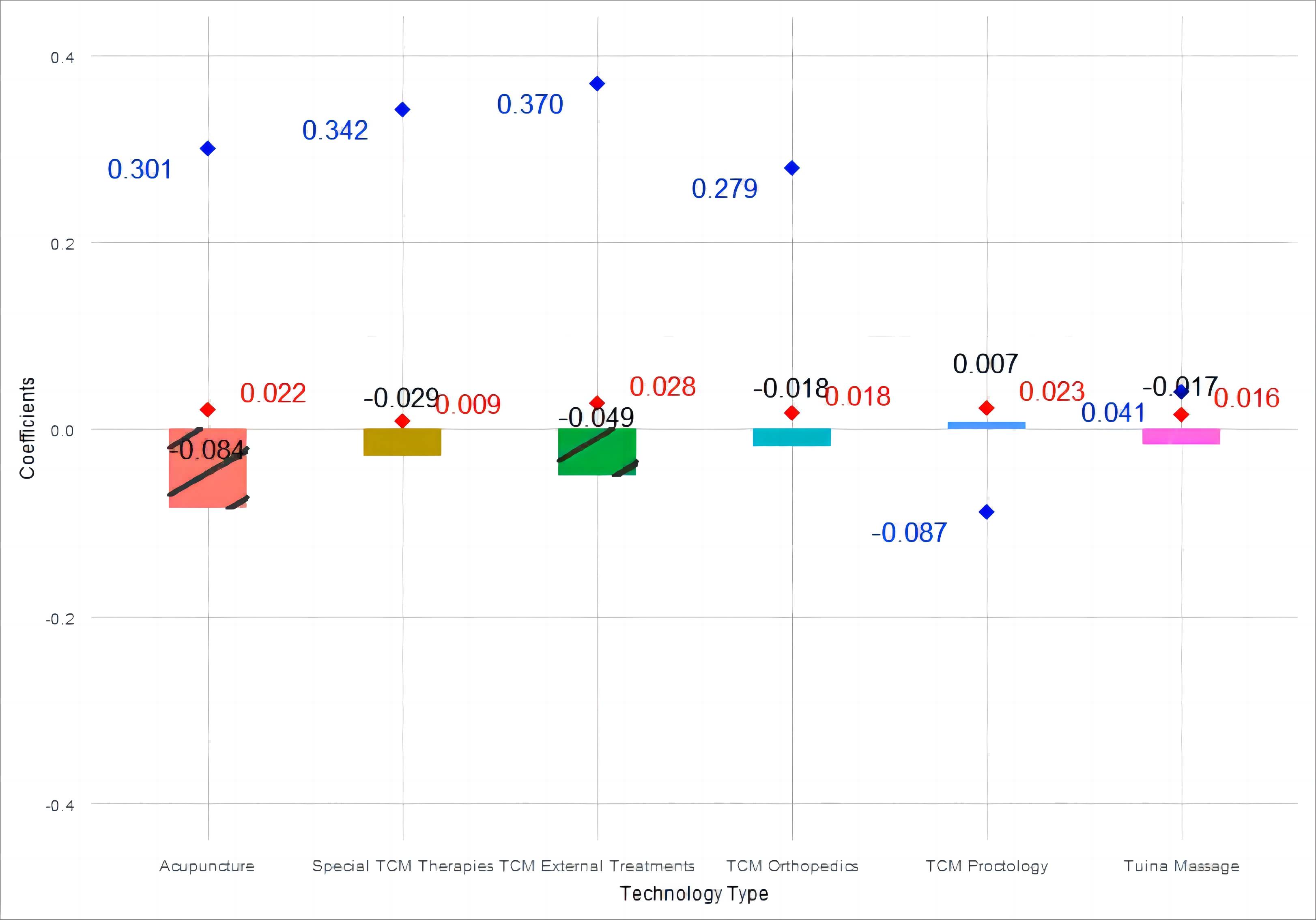

Supplement: Supplementary file 1 [file Datasheet1.zip › Supplementary/Supplementary-FIGURE2.jpg]

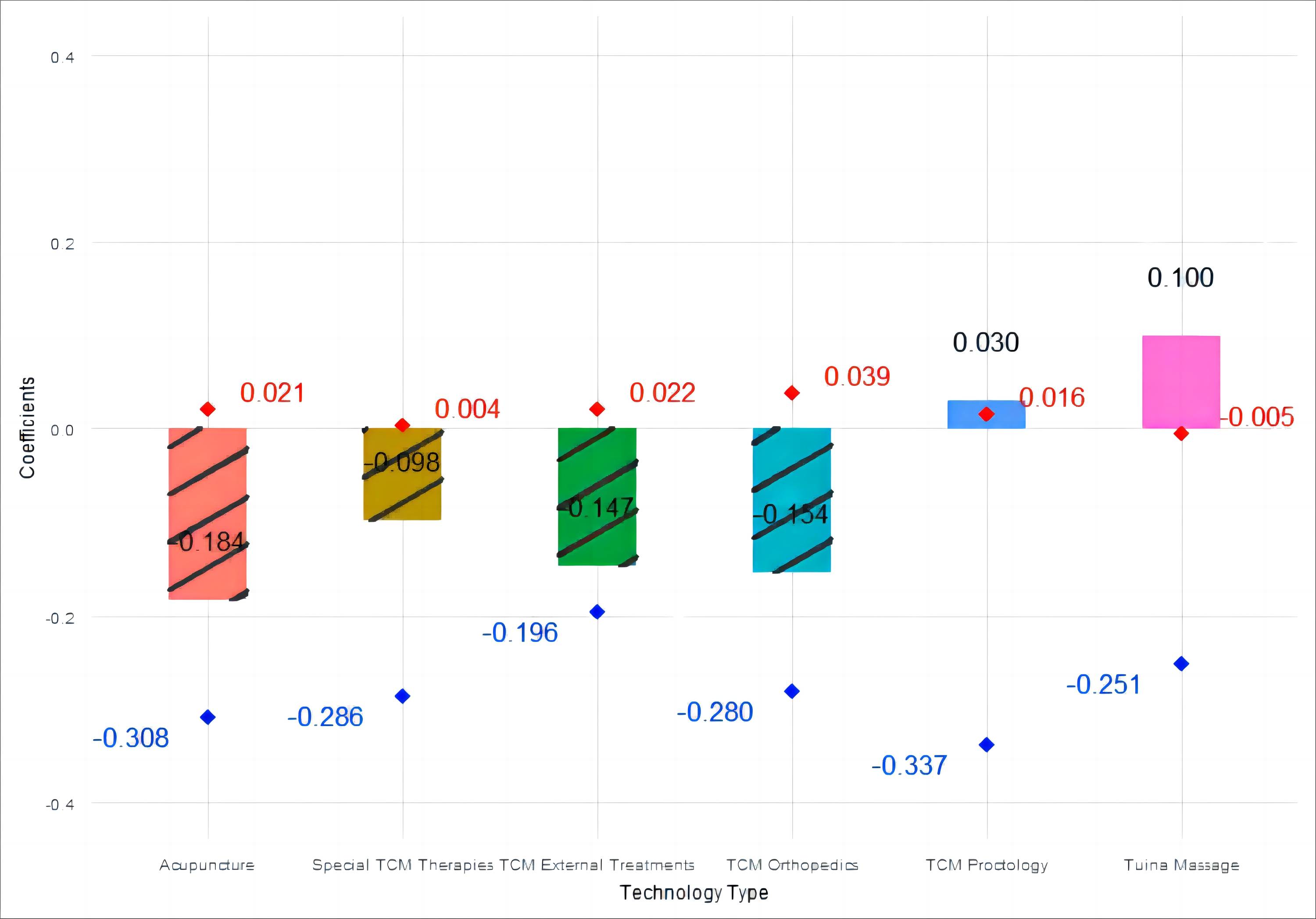

Supplement: Supplementary file 1 [file Datasheet1.zip › Supplementary/Supplementary-FIGURE3.jpg]

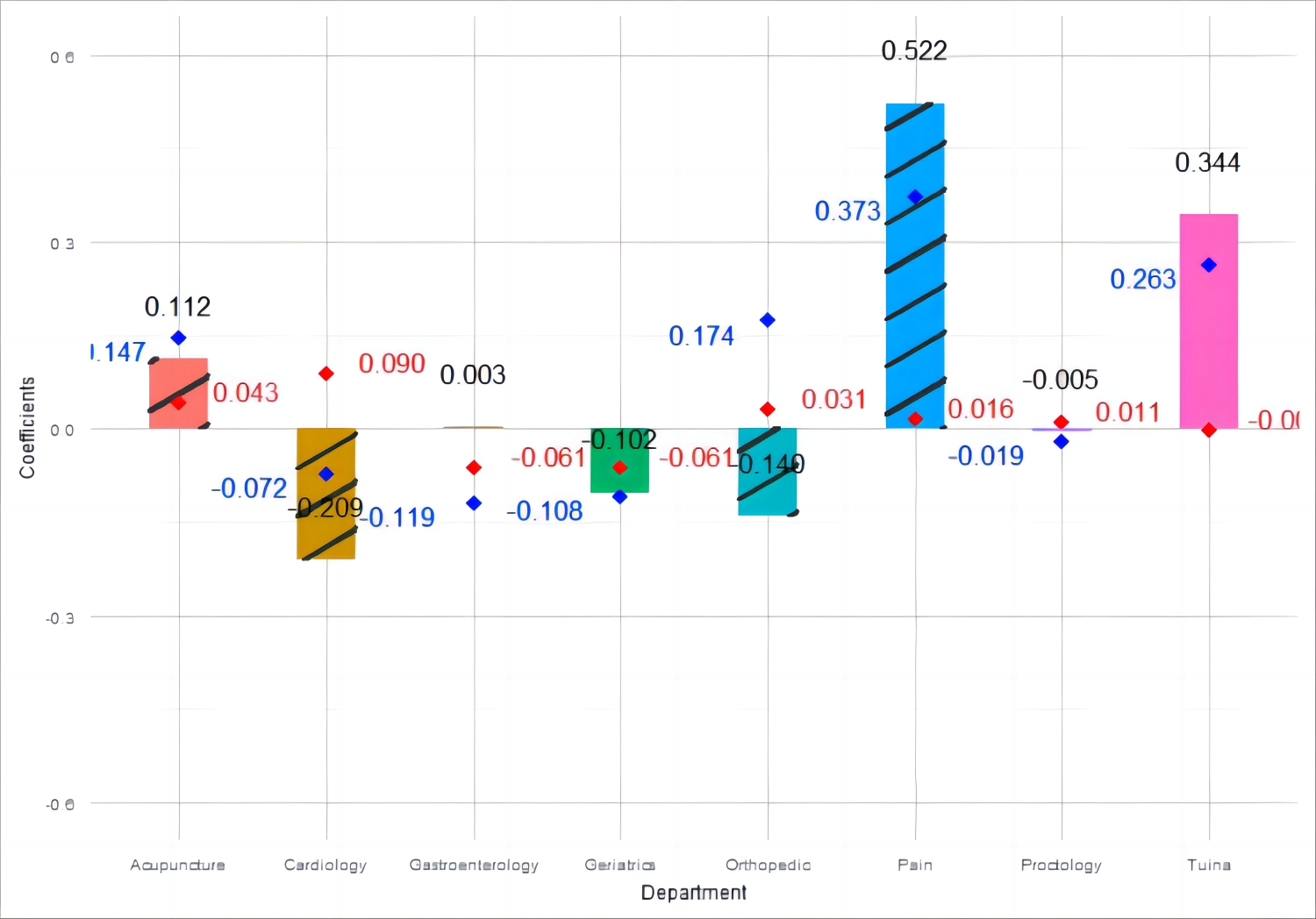

Supplement: Supplementary file 1 [file Datasheet1.zip › Supplementary/Supplementary-FIGURE4.jpg]

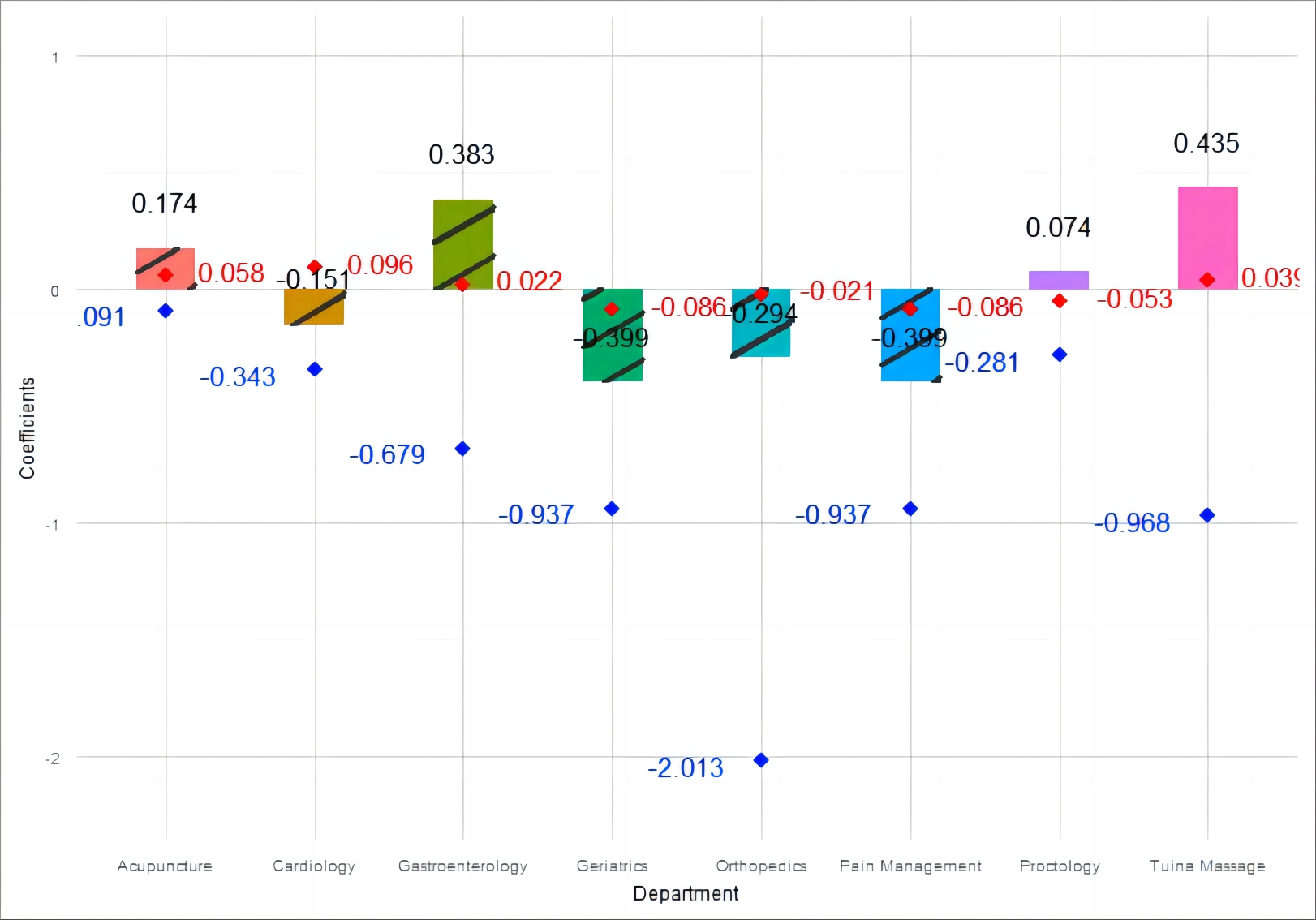

Supplement: Supplementary file 1 [file Datasheet1.zip › Supplementary/Supplementary-FIGURE5.jpg]
