# Supplementary material for: The impact of DRG reimbursement system on appropriate techniques of traditional Chinese medicine—evidence from pilot cities with traditional Chinese medicine hospitals in China
Source: Front Health Serv. 2025 Aug 4;5:1441482. doi: 10.3389/frhs.2025.1441482 (PMC12358404; doi:10.3389/frhs.2025.1441482)
Supplement: Supplementary file 2 [file Table1.doc]

Supplementary Material

# Supplementary Data

Data screening rules for the experimental group (Appropriate technology of TCM) and the control group (other group) are as follows:

(1). Criteria for selecting suitable traditional Chinese medicine techniques: Treatments primarily with traditional Chinese medicine, with treatment costs exceeding surgical costs by more than onefold, and surgical operations include traditional Chinese medicine techniques. Exclude cases primarily treated with Western medicine.

(2). Criteria for selecting control group: Mainly select cases primarily treated with Western surgical operations, with costs exceeding those of traditional Chinese medicine by more than onefold, and possess Western surgical operation codes. Exclude cases primarily treated with traditional Chinese medicine.

(3). Case matching: Ensure that the two groups of cases are comparable in personal basic characteristics (age, gender).

(4). Data screening: Filter out medication costs to ensure they are less than 1fold of the treatment cost.

(5). Data processing: Process extreme values in length of stay and costs, eliminate outliers, and ensure data is scientifically accurate and accurate.

# Supplementary Figures

## Supplementary Figures

FIGURE 1 The flowchart of this study (the last one)

Table 6-9: Results presented in the format of Figure 2-5.


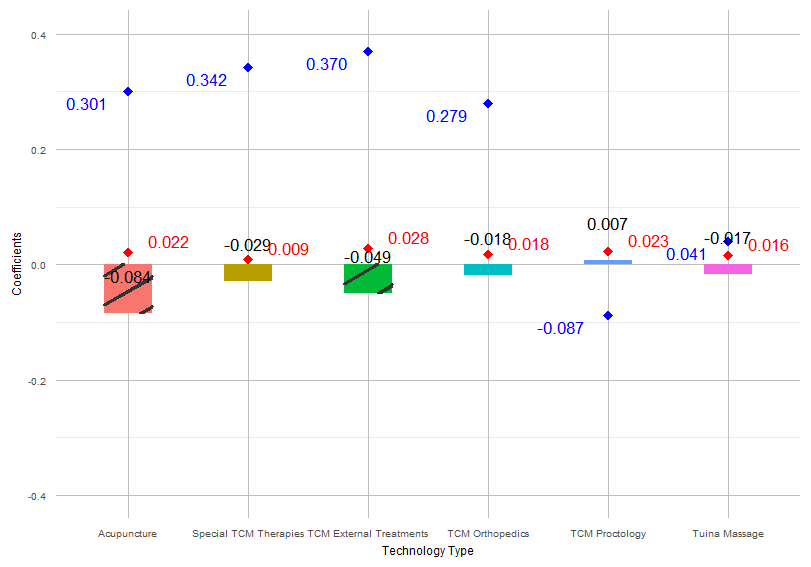


Figure 2 The impact of DRG payment system implementation on the total cost of using various TCM appropriate technologies


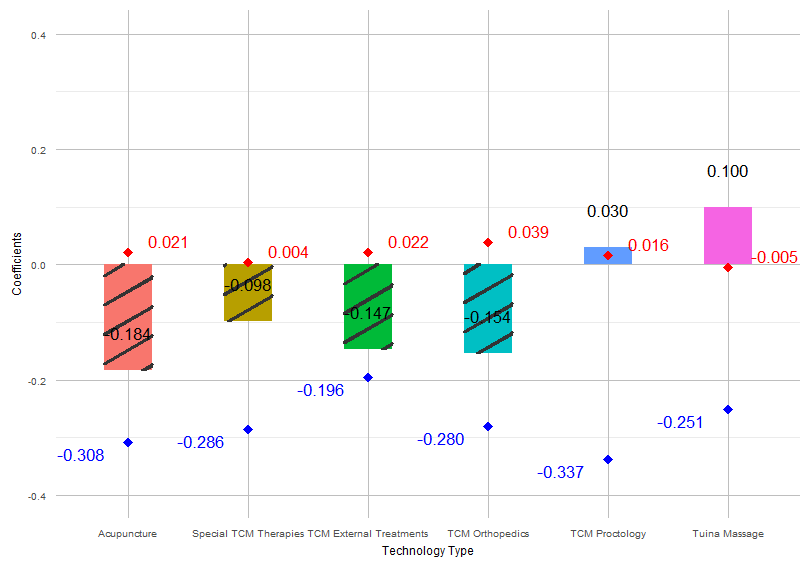


FIGURE 3 The impact of DRG payment system implementation on the cost of using various TCM appropriate technologies


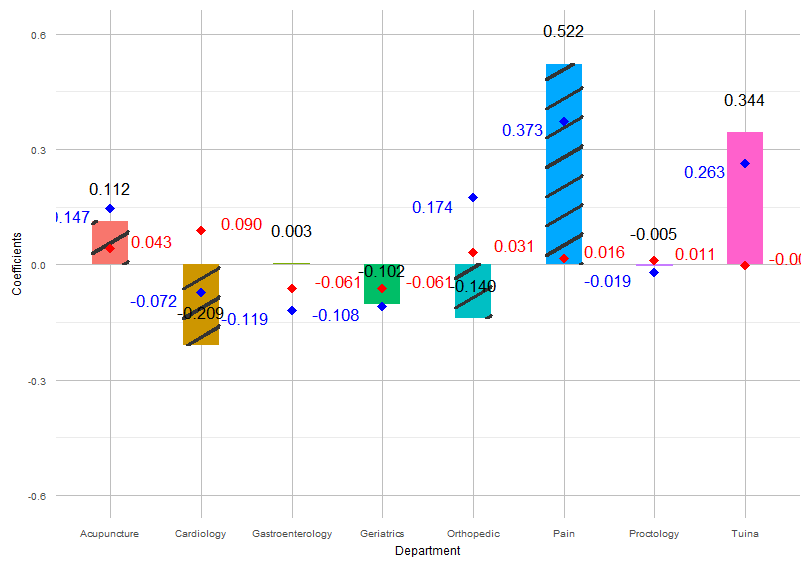


FIGURE 4 The impact of DRG payment system on departmental total expenses


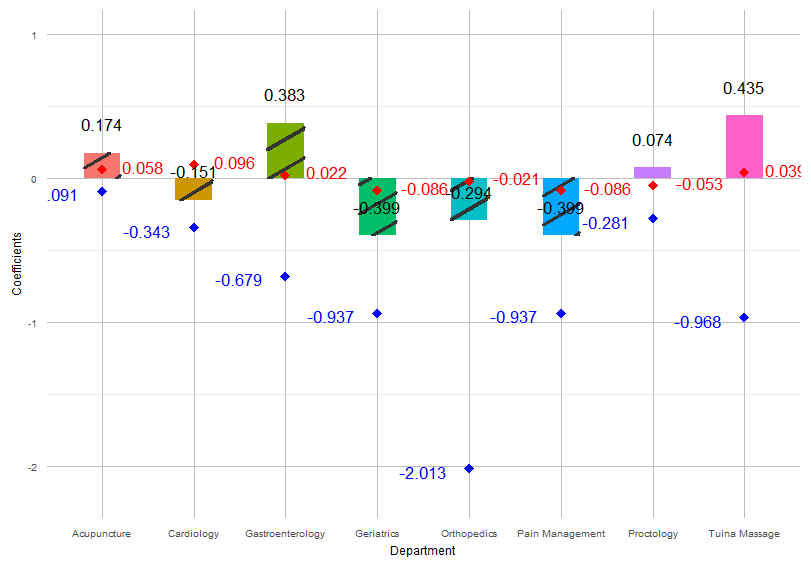


FIGURE 5 Impact of DRG payment system implementation on the cost of Chinese medicine appropriate technologies in departments


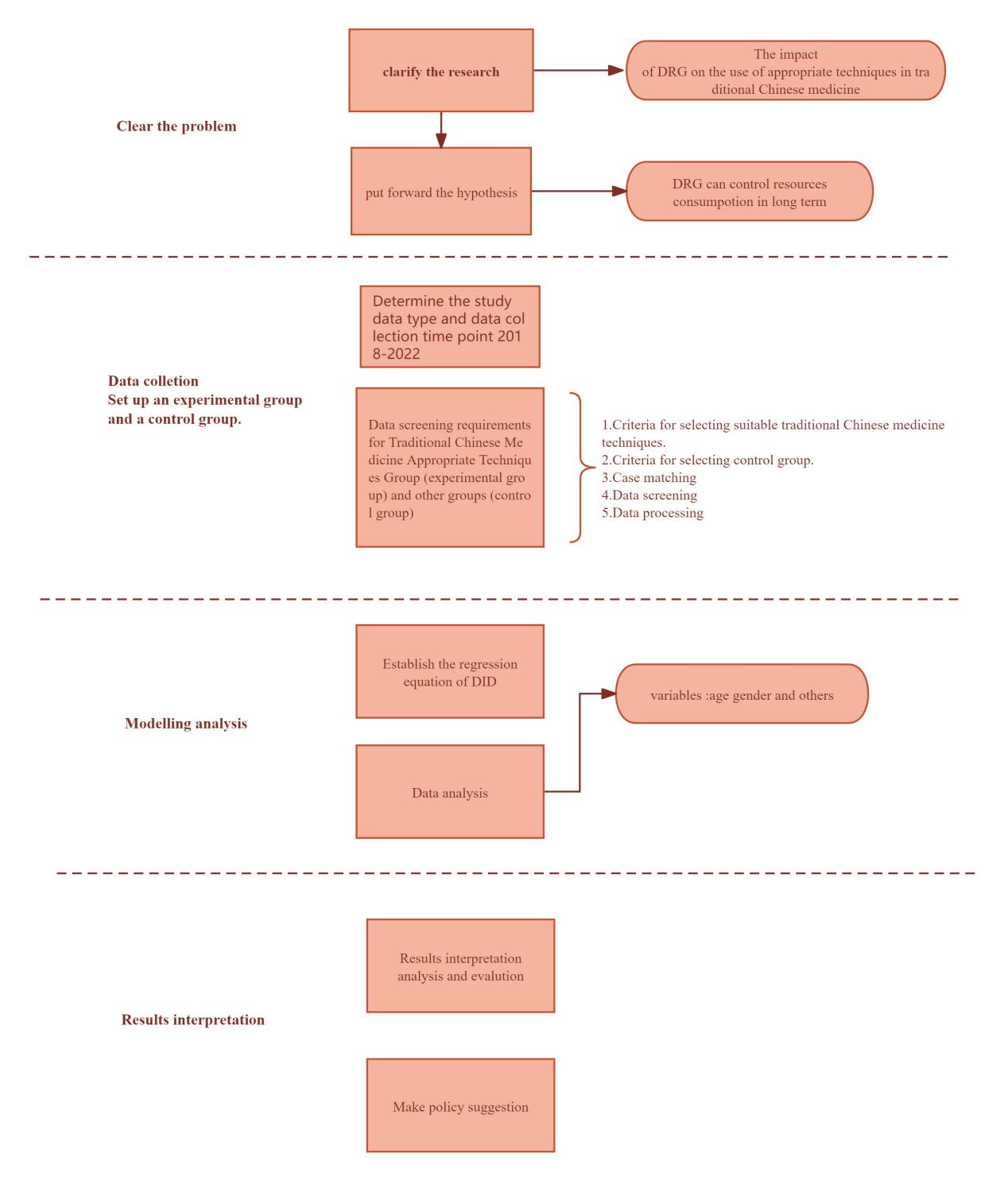


FIGURE1 The flowchart of this study
